# Predicting the Current Potential Suitable Habitat of *Pinus tabuliformis* in China Using Multi‐Source Environmental Data and Ensemble Learning Models

**DOI:** 10.1002/ece3.74290

**Published:** 2026-09-10

**Authors:** Haodong Ding, Jie Meng, Yaru Zhang, Ying Yang, Wei Deng, Duanyang Xu

**Affiliations:** ^1^ Institute of Geographic Sciences and Natural Resources Research Chinese Academy of Sciences Beijing China; ^2^ University of Chinese Academy of Sciences Beijing China; ^3^ School of Geography and Information Engineering China University of Geosciences Wuhan China

**Keywords:** ensemble learning model, generalized additive model, MaxEnt, *Pinus tabuliformis*, random forest, species distribution model

## Abstract

*Pinus tabuliformis* is an endemic tree species in China that supports windbreak and sand fixation, soil and water conservation, and ecosystem stability. However, some stands experience growth decline and degradation, especially in arid and semi‐arid regions with constrained regeneration. Identifying key environmental drivers and suitable habitats is therefore important for restoration and management. Because species distribution model outputs vary among algorithms, we developed a spatially validated stacking framework integrating maximum entropy (MaxEnt), random forest (RF), and a generalized additive model (GAM) using multi‐source environmental data. Predictors were screened using variance inflation factor (VIF < 5) and correlation threshold (|*r*| < 0.8). The results showed that: (1) Seasonal hydrothermal conditions and topography primarily shaped the current potential distribution of *P. tabuliformis* . BIO9, DEM, and BIO18 were the leading predictors, with BIO9 ranking first across all models and highest suitability occurring at −10°C to 5°C. (2) Under five‐fold spatial block cross‐validation, RF achieved the highest mean AUC, TSS, and width‐normalized partial AUC values of 0.973 ± 0.031, 0.726 ± 0.140, and 0.574 ± 0.245, respectively. The stacking ensemble showed numerically similar AUC and TSS values but did not outperform RF. No pairwise performance differences remained statistically significant after Holm correction. (3) Highly suitable habitats were concentrated in low‐ to mid‐elevation mountains and hills of northern and north‐central China, especially the Loess Plateau and adjacent areas, with the Qinling Mountains forming a southern transition zone. The ensemble predicted a total suitable habitat area of 28.45 × 10^5^ km^2^ (95% CI: 25.23–31.42 × 10^5^ km^2^), with a suitability‐weighted centroid at approximately 34.50° N and 110.72° E. Overall, stacking integrated structurally different algorithms into a synthesized spatial estimate supporting field validation, conservation, restoration, afforestation zoning, and sustainable management.

## Graphical abstract

This study integrated multi‐source environmental variables and species occurrence records to predict the current potential suitable habitat of *Pinus tabuliformis* in China using MaxEnt, Random Forest (RF), generalized additive model (GAM), and a stacking ensemble model. Mean temperature of the driest quarter, elevation, and precipitation of the warmest quarter were identified as the principal environmental predictors of its current potential distribution. Under five‐fold spatial block cross‐validation, RF achieved the highest mean AUC, TSS, and width‐normalized partial AUC, whereas the stacking ensemble showed numerically similar AUC and TSS values but did not outperform RF. No pairwise performance differences remained statistically significant after Holm correction. Highly suitable habitats were concentrated mainly in northern and north‐central China, particularly across the Loess Plateau and adjacent mountainous and hilly regions, while the Qinling Mountains formed an important southern transition zone.

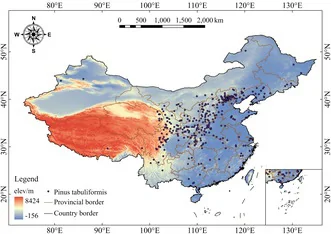

## 1 Introduction

*Pinus tabuliformis* , belonging to the family Pinaceae and the genus *Pinus*, is an important tree species endemic to China. It is widely distributed in northern China, including North China, Northeast China, Northwest China, the Loess Plateau, the Shandong Peninsula, and areas north of the Qinling Mountains. It commonly occurs on sunny mountain and hill slopes, rocky sites, and relatively infertile soils (Liu and Wang 2024; Zhang et al. 2006). As one of the dominant tree species in northern forests, *P. tabuliformis* plays an important ecological role in soil and water conservation, windbreak and sand fixation, ecological security maintenance, and vegetation restoration, and also has ornamental value (Chen et al. 2021). Although *P. tabuliformis* is a cold‐ and drought‐tolerant, deep‐rooted conifer, its establishment, regeneration, and growth remain sensitive to seasonal water availability, temperature, and topographically mediated site conditions. These trait–environment relationships provide an ecological basis for including climatic, topographic, soil, and hydrological variables as predictors of habitat suitability. Regional‐scale studies have documented substantial growth decline of *P. tabuliformis* under increasingly warm and dry conditions. For example, Zhao et al. (2021) documented growth decline in *P. tabuliformis* plantations in the drier southwestern part of the semi‐arid region of Northeast China, where warming intensified drought stress and water limitation. In addition, a synthesis of tree‐ring width chronologies from 60 sites across the major geomorphological units of the Loess Plateau showed significant declines in radial growth after 1960, with the strongest decline in the southern region (*R* = −0.477, *p* < 0.01) and significant negative trends also occurring in the eastern and western regions (*R* = −0.325 to −0.353, *p* < 0.01) (Li et al. 2026). These findings indicate that even this drought‐tolerant species can experience widespread growth decline under increasingly unfavorable hydrothermal conditions. Therefore, *P. tabuliformis* was selected as the target species because of its ecological importance, wide distribution in northern China, sensitivity to environmental gradients, and practical relevance to conservation planning and afforestation zoning. Identifying the key environmental factors affecting the distribution of *P. tabuliformis* and predicting its current potential suitable habitats are of great significance for conservation planning, ecological restoration, afforestation zoning, and sustainable forest management.

Species distribution models (SDMs) are important tools for inferring species niches and predicting potential distributions across space and time based on statistical relationships between species occurrence records and environmental factors (Martínez‐Minaya et al. 2018). With the development of statistical methods and machine learning techniques, various SDM frameworks have been developed, including the Maximum Entropy model (MaxEnt), BIOCLIM, Random Forest (RF), and Generalized Additive Model (GAM). These models have been widely applied in tree species distribution prediction, climate change response assessment, habitat suitability analysis, and conservation priority identification (Phillips et al. 2006; Zhao et al. 2022; Hastie and Tibshirani 2017). Studies on the distribution pattern of *P. tabuliformis* have also made progress. For example, Liu and Wang (2024) used the MaxEnt model to analyze changes in the suitable distribution area of *P. tabuliformis* plantations in China under climate change and found that the suitable area tended to shift northward, with precipitation of the warmest quarter and temperature factors being the main limiting variables. Li et al. (2023) used MaxEnt to simulate the potential suitable distribution of *P. tabuliformis* in the Yiluo River Basin and found that annual precipitation, soil type, and elevation were key drivers, with suitable habitats mainly concentrated in the central and southern mountainous areas of the basin. Although these studies have provided important references for understanding the suitable distribution pattern of *P. tabuliformis* , several limitations remain. On the one hand, different species distribution modeling algorithms vary in their representation of nonlinear responses, variable interactions, niche boundaries, and probability outputs. Consequently, results derived from a single algorithm may reflect the characteristics of a particular model structure, making it difficult to assess whether the importance and response patterns of key environmental predictors are consistently supported across algorithms (Merow et al. 2013; Norberg et al. 2019). On the other hand, similar values of conventional evaluation metrics do not necessarily correspond to similar spatial predictions. Different algorithms may still produce substantial differences in the predicted extent of suitable habitat, the allocation of area among suitability classes, and the geographic concentration of habitat suitability. Model evaluation may also be overly optimistic when spatial dependence between training and testing records is not adequately controlled. Accordingly, a spatially validated stacking ensemble framework provides an appropriate approach for integrating predictions from structurally different algorithms, comparing the predictive performance, environmental‐response patterns, and spatial outputs of the base and ensemble models under a common validation scheme, and generating a synthesized estimate of the current potential suitable habitat of *P. tabuliformis* (Bi et al. 2013; Araújo and New 2007).

MaxEnt, RF, and GAM represent three mainstream SDM paradigms based on the maximum entropy principle, ensemble tree methods, and additive smooth regression, respectively. They differ in assumptions about variable responses, nonlinear fitting ability, and interpretability. MaxEnt is specifically designed for presence‐only data and shows strong robustness for rare species and incompletely sampled data (Phillips et al. 2006). RF can effectively capture high‐order interactions and complex nonlinear relationships among variables and is relatively insensitive to outliers (Breiman 2001; Cutler et al. 2007). GAM uses smooth functions to fit response relationships while retaining an additive model structure, which facilitates ecological interpretation of individual variable effects (Yee and Mitchell 1991; Wood 2017). These three models can approximate the realized niche characteristics of *P. tabuliformis* from different perspectives and thus complement one another.

In response to the above research gaps, this study used distribution records of *P. tabuliformis* and multiple environmental variables to predict its current potential suitable habitats. Specifically, we tested three hypotheses. First, seasonal hydrothermal conditions and topography, particularly mean temperature of the driest quarter (BIO9), precipitation of the warmest quarter (BIO18), and elevation, would be major predictors of habitat suitability. Second, we hypothesized that the stacking ensemble would not necessarily outperform the individual models across all evaluation metrics under spatially independent validation. Third, highly suitable habitats would be concentrated mainly in low‐ to mid‐elevation mountainous and hilly regions of northern and north‐central China, particularly across the Loess Plateau and adjacent areas.

To test these hypotheses, we first screened environmental variables using variance inflation factor and correlation analyzes, and then constructed MaxEnt, RF, GAM, and stacking ensemble models. Predictive performance was evaluated under five‐fold spatial block cross‐validation using the area under the receiver operating characteristic curve (AUC), the true skill statistic (TSS), and partial AUC. On this basis, ArcGIS was used to classify suitable habitats and conduct comparison of suitability‐weighted centroids among models. Partial dependence plots were further used to identify the key factors affecting the distribution of *P. tabuliformis* , and the spatial patterns and centroid differences among different models were analyzed. The results aim to provide a scientific basis for the conservation, restoration, and sustainable management of *P. tabuliformis* .

## 2 Materials and Methods

### 2.1 Study Area

The study area covered the whole territory of China. Although *P. tabuliformis* is mainly distributed in northern China, a national modeling extent was used to evaluate its potential environmental suitability across the full climatic and topographic gradients of China without restricting predictions a priori to its currently known distribution. China has complex topography, with an overall west‐high and east‐low terrain pattern and diverse landforms, including plateaus, mountains, hills, basins, and plains. This broad topographic gradient is commonly described as three major terrestrial terrain steps. The first step is dominated by the Qinghai–Tibet Plateau, with an average elevation above 4000 m. The second step, generally at elevations of approximately 1000–2000 m, includes major plateaus and basins such as the Inner Mongolia Plateau, Loess Plateau, Tarim Basin, and Sichuan Basin. East of the Greater Khingan–Taihang–Wushan–Xuefeng mountain line, the terrain descends to the third step, which is dominated by lower‐elevation plains and hills extending toward the eastern coast (Xie et al. 2022). The potential distribution region of *P. tabuliformis* is mainly associated with northern China, including mountainous and hilly areas north of the Qinling Mountains, the Taihang Mountains, the Loess Plateau, the Yan Mountains, and the western Liaoning Mountains (Liu and Wang 2024). These areas contain strong gradients in elevation, slope, aspect, and drainage conditions, which can influence local temperature, moisture availability, soil development, and vegetation distribution.

China also spans broad climatic gradients, ranging from humid and semi‐humid regions in eastern and southeastern China to arid and semi‐arid regions in northern and northwestern China (Ma et al. 2019). The distribution region of *P. tabuliformis* is mainly located in temperate and warm‐temperate zones, where seasonal differences in temperature and precipitation are pronounced. As a light‐demanding and deep‐rooted conifer, *P. tabuliformis* is generally adapted to cold and relatively dry environments, but its regeneration and growth still depend on an appropriate seasonal balance between temperature and moisture. In the Qinling Mountains, which form an important southern marginal and transitional zone, light conditions, soil conditions, and regeneration dynamics also influence the local distribution and persistence of *P. tabuliformis* populations (Yang et al. 2023). Therefore, the strong climatic and topographic heterogeneity of the study area provides an appropriate environmental gradient for assessing the current potential suitable habitats and key environmental constraints of *P. tabuliformis* .

### 2.2 Distribution Data of *Pinus tabuliformis*

**FIGURE 1:**
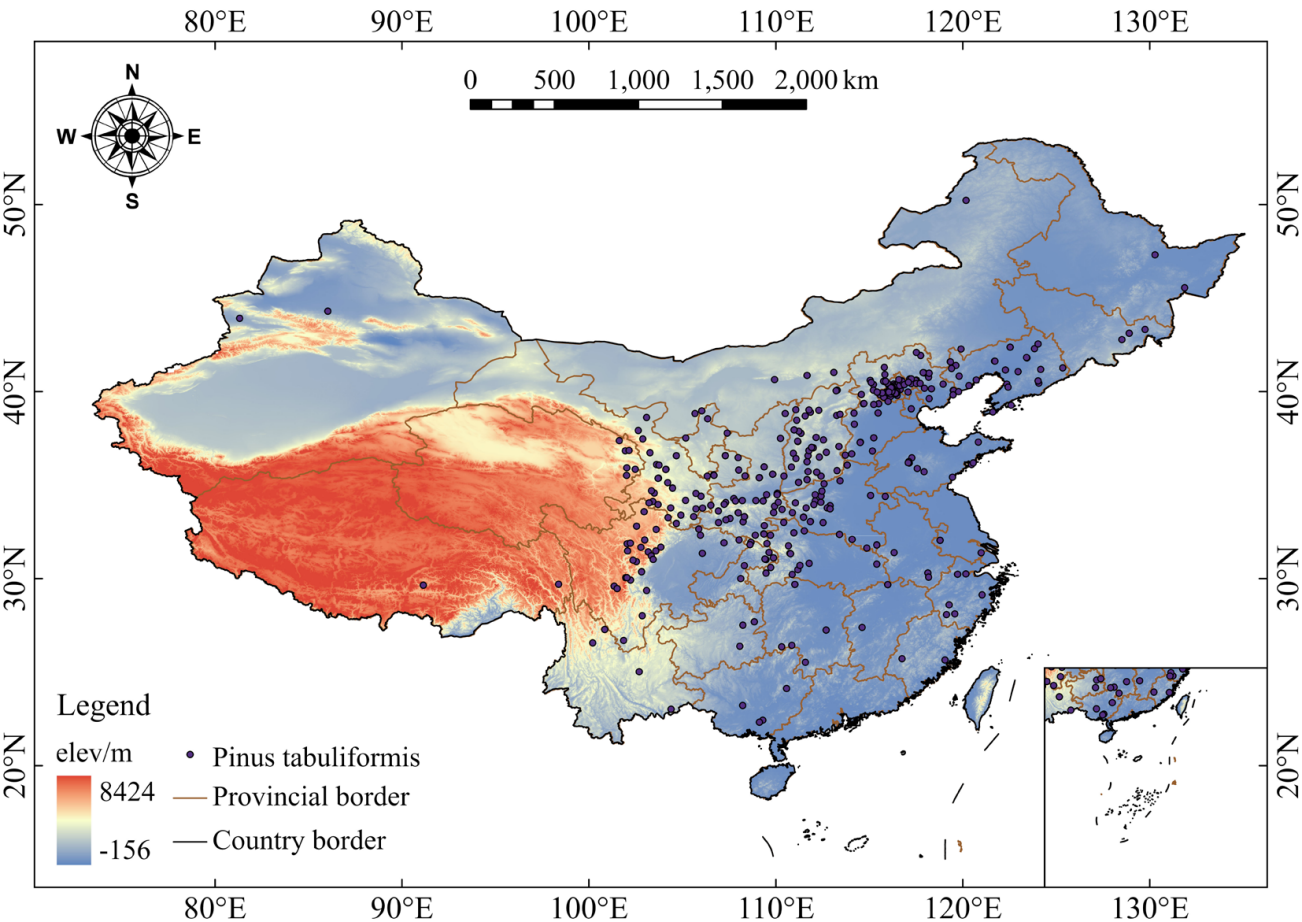
Study area and occurrence records of *Pinus tabuliformis* used for species distribution modeling.

The distribution records of *Pinus tabuliformis* were obtained from the Global Biodiversity Information Facility (GBIF, https://www.gbif.org/) and were downloaded on 25 November 2024. A total of 1216 georeferenced occurrence records within China were collected. The GBIF records were not independently cross‐referenced with herbarium databases or field‐survey records. Quality control therefore focused on retaining georeferenced records within China, duplicate removal, and spatial thinning. Because GBIF records are compiled from heterogeneous and non‐standardized surveys, they may be spatially biased toward accessible and more frequently surveyed areas. Direct quantification of accessibility and survey effort bias was not possible because standardized information on survey routes and sampling effort was unavailable. To reduce the spatial clustering and redundancy associated with such uneven sampling, the records were spatially thinned after duplicate removal using the “Spatially Rarefy Occurrence Data for SDMs” tool in SDM Toolbox, with no more than one record retained within each 1 km × 1 km grid cell. The thinning resolution was selected to match the spatial resolution of the environmental predictors and thereby reduce spatial redundancy and potential model overfitting. This procedure resulted in 389 valid occurrence records (Figure 1), primarily reflecting the strong spatial clustering of the original GBIF data. Of these records, 253 (65.0%) were recorded during 1950–1989 and 77 (19.8%) during 2000–2024, with the available year information spanning 1903–2024. Although thinning may have excluded some ecologically meaningful variation at scales finer than 1 km, such fine‐scale heterogeneity could not be explicitly represented by the environmental predictors used in this study. The retained records were converted to CSV format for subsequent modeling.

### 2.3 Environmental Data

A total of 39 environmental variables were selected in this study (Table 1). All variables were uniformly reprojected, clipped, and resampled to a spatial resolution of 1 km to ensure consistency in coordinate system, spatial resolution, and study extent. Considering the potential strong collinearity among environmental factors and to avoid its interference with model results, the variance inflation factor (VIF) was first calculated iteratively, and variables with VIF values greater than 5 were gradually removed to reduce multicollinearity. Subsequently, a Pearson correlation coefficient matrix was used to further remove redundant variables with absolute correlation coefficients greater than 0.8 (Dormann et al. 2013; Sillero and Barbosa 2021). Finally, 11 environmental variables were retained for modeling, including BIO18 (precipitation of the warmest quarter), BIO19 (precipitation of the coldest quarter), BIO9 (mean temperature of the driest quarter), DEM, AWC (available water capacity), LBGC (long‐term bare ground cover), HAND (height above nearest drainage), aspect, slope, Upa (upstream contributing area), and Snow_05 (annual mean of monthly P05 snow‐cover fraction). For each month, P05 represents the 5th percentile of daily snow‐cover fraction within that month. The monthly P05 layers were subsequently averaged across months to obtain Snow_05, which summarizes the lower‐end snow‐cover condition over the year. Higher Snow_05 values indicate that a relatively larger fraction of the ground remains snow covered even during comparatively low‐snow periods, thereby reflecting more persistent snow‐cover conditions.

**TABLE 1:** Environmental data sources table.

Category	Data type	Data source
Climate variables	Annual snow‐cover fraction layers (**Snow_05**, Snow_50, and Snow_95), calculated as the annual means of monthly P05, P50, and P95 layers, respectively	https://doi.org/10.5281/zenodo.5774953
	Mean land surface temperature	https://www.ncdc.ac.cn/portal/
	Mean precipitation	https://www.worldclim.org
	Mean minimum, mean, and maximum air temperature	https://www.worldclim.org
	Mean wind speed	https://www.worldclim.org
	Mean vapor pressure	https://www.worldclim.org
	Direct and diffuse solar radiation	https://globalsolaratlas.info/download
	Mean cloud cover	https://www.earthenv.org/cloud
	Nineteen bioclimatic variables from WorldClim (retained: **BIO9, BIO18, and BIO19**)	https://www.worldclim.org
Topographic and land surface variables	**Long‐term bare ground cover (LBGC)**	https://glad.umd.edu/dataset/global‐2010‐bare‐ground‐30‐m
	**Digital elevation model (DEM)**	https://www.worldclim.org
	**Slope** and **aspect**	Derived from DEM
Hydrological variables	**Height above nearest drainage (HAND)** and **upstream contributing area (Upa)**	http://hydro.iis.u‐tokyo.ac.jp/~yamadai/MERIT_Hydro/
Soil variables	**Available water capacity (AWC)**	https://www.fao.org/

*Note:* The 11 variables retained for model construction are shown in bold.

Because GAM and Random Forest models require both presence and absence data during model training, whereas only species presence records were available in this study, 1000 pseudo‐absence points were generated using the “biomod2” package in R. Given that the method used to generate pseudo‐absence points can significantly affect model performance (Barbet‐Massin et al. 2012; Phillips et al. 2009), the surface range envelope (SRE) strategy was applied. For each environmental predictor, SRE defined the environmental range represented by the occurrence records using the 2.5th and 97.5th percentiles, and background cells outside the resulting environmental envelope were considered eligible for pseudo‐absence selection. A total of 1000 pseudo‐absence points were randomly sampled from these eligible cells. This number was selected to provide sufficient representation of the environmental background while maintaining a reasonable balance between presence and pseudo‐absence samples. Unlike unrestricted random‐background sampling, SRE applies a species‐specific environmental constraint based on the observed occurrence records; compared with general environmental stratification, it does not require an arbitrary division of the entire environmental space. The generated pseudo‐absence points were then combined with the occurrence records to construct the final modeling dataset in CSV format.

### 2.4 Research Methods

**FIGURE 2:**
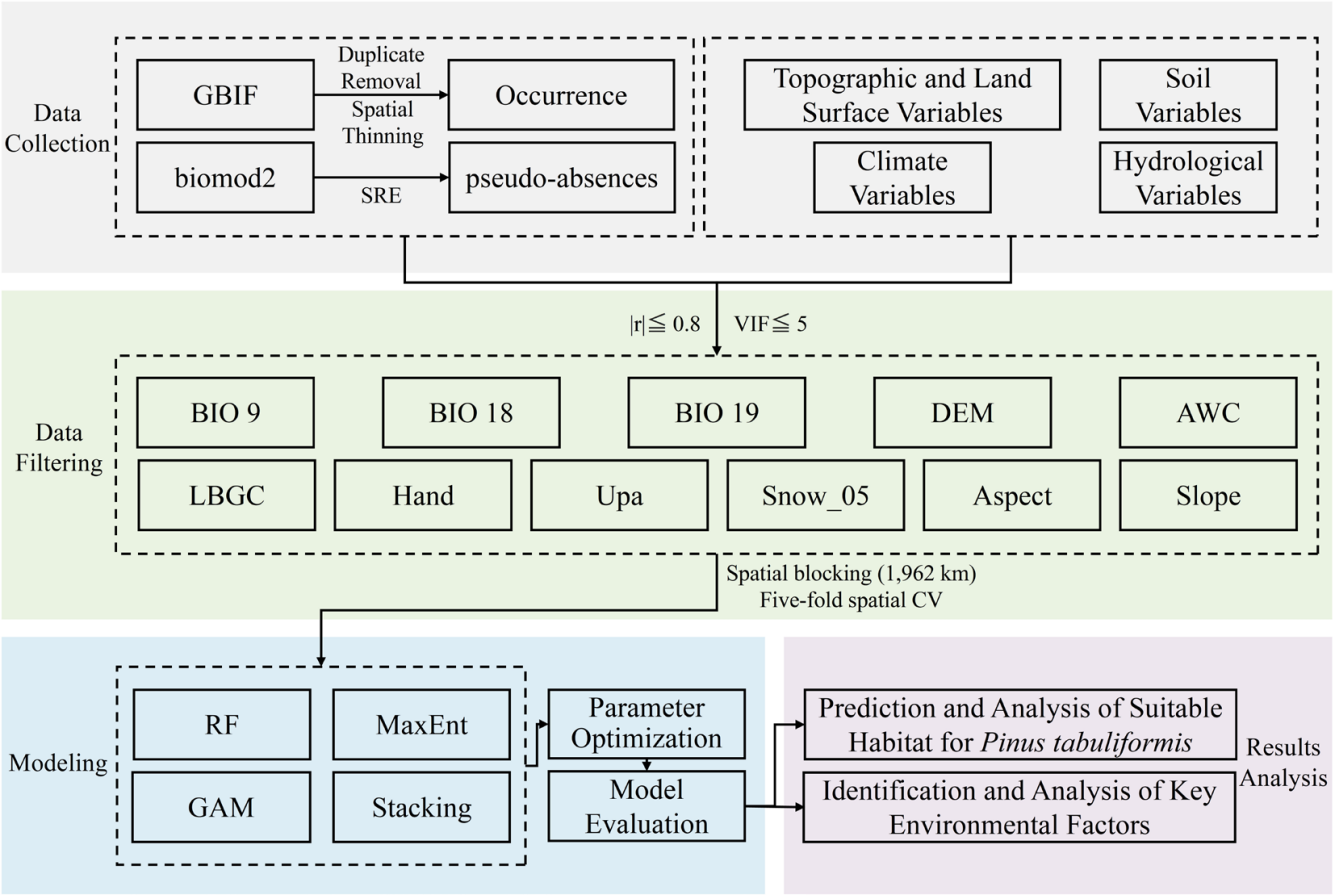
Technical workflow for predicting the potential suitable habitat of *Pinus tabuliformis* based on multi‐source environmental data and ensemble learning models.

This study focused on the current potential suitable habitat of *P. tabuliformis*. Therefore, all model training, evaluation, variable importance analysis, response‐curve calculation, and spatial predictions were conducted under current environmental conditions. The technical workflow consisted of four main stages: data collection, data filtering, modeling, and results analysis (Figure 2). First, during the data preparation stage, four categories of environmental data—topographic and land surface variables, soil variables, climatic variables, and hydrological variables—were collected, together with species presence records and pseudo‐absence data. Correlation and collinearity analyzes were then performed to select 11 environmental variables for model construction. During the modeling stage, MaxEnt, RF, GAM, and Stacking models were constructed and trained, and systematic hyperparameter optimization was performed to improve model performance. The hyperparameters of the three base learners and the logistic‐regression meta‐learner were optimized using stratified 10‐fold internal cross‐validation. Model performance was subsequently evaluated using the corresponding held‐out spatial folds. In the results analysis stage, the potential suitable habitats of *P. tabuliformis* were predicted and analyzed, and the key environmental factors influencing its distribution were identified and evaluated.

#### 2.4.1 Model Construction and Optimization

Model construction was conducted within an outer five‐fold spatial block cross‐validation framework, with stratified 10‐fold cross‐validation used for internal hyperparameter tuning. Maximum entropy (MaxEnt), random forest (RF), and generalized additive models (GAMs) were used as base learners. To reduce spatial dependence between training and testing samples, the presence and pseudo‐absence records were partitioned using spatial block cross‐validation rather than random splitting (Koldasbayeva and Zaytsev 2025). The block width was determined from the spatial autocorrelation range of the binary response data comprising presence and pseudo‐absence records, using the cv_spatial_autocor function in the R package blockCV, yielding a value of 1962 km. This relatively large value was data‐driven rather than arbitrarily selected and reflects the broad‐scale spatial autocorrelation structure of the binary response data across the national modeling extent. Using this width, square blocks were generated and assigned to five folds with the cv_spatial function. In each outer iteration, four folds constituted the training set and were used for hyperparameter optimization and model fitting, whereas the remaining fold constituted a spatially separated test set used exclusively for model performance evaluation and was not involved in any tuning or fitting procedures, including meta‐learner training. Across the five outer iterations, each spatial fold served as the test set once.

Considering that default parameter settings may lead to insufficient model performance or biased results, systematic hyperparameter optimization and validation were conducted for each model (Montoya‐Jiménez et al. 2025). RF, MaxEnt, GAM, and the stacking model used the same stratified 10‐fold internal cross‐validation partitions and random seed. For each base learner, candidate parameter settings were evaluated according to mean internal AUC. The best‐performing setting was then used to refit the model on the complete outer training dataset.

For RF, the number of trees was fixed at 500, and the number of predictors considered at each split was set to one‐third of the total number of predictors (max_features = 4). Maximum tree depth was evaluated at 5, 10, 15, and 20, together with an unrestricted‐depth option, whereas the minimum number of samples per terminal leaf was evaluated at 1, 3, 5, 7, 9, and 11.

For GAM, a logistic generalized additive model with a logit link was fitted, with an additive penalized B‐spline smooth term specified for each continuous predictor. Fifty candidate parameter combinations were randomly sampled. The number of spline basis functions ranged from 5 to 9, and the smoothing penalty parameter (λ) was sampled from a log‐uniform distribution between 1 and 1000. Quadratic B‐spline basis functions and an intercept were used (Wood 2017).

For MaxEnt, five feature‐class (FC) combinations were evaluated: linear (L), linear–quadratic (LQ), hinge (H), linear–quadratic–hinge (LQH), and linear–quadratic–hinge–product (LQHP). Each combination was paired with regularization multipliers (RM) ranging from 0.5 to 4.0 at intervals of 0.5, resulting in 40 candidate settings.

The optimized RF, MaxEnt, and GAM models were integrated using a stacking framework with logistic regression as the meta‐learner. The selected hyperparameters of the base learners were retained during stacking and were not reoptimized. Within each outer training dataset, stratified 10‐fold cross‐validation was used to generate out‐of‐fold probabilities of presence from the three base learners. Each training sample therefore received one probability from each model that had not been fitted using that sample, and these probabilities were used as meta‐features. The final stacking probability was calculated as follows:

(1) $$p_{\text{ensemble}}={\text{logit}}^{-1}\left(\beta_{0}+\beta_{\mathrm{RF}}p_{\mathrm{RF}}+\beta_{\text{MaxEnt}}p_{\text{MaxEnt}}+\beta_{\mathrm{GAM}}p_{\mathrm{GAM}}\right)$$

where $p_{\mathrm{RF}}$, $p_{\text{MaxEnt}}$, and $p_{\mathrm{GAM}}$ are the probabilities of presence predicted by the three base learners; $\beta_{0}$ is the intercept; and the β terms are coefficients estimated by the logistic‐regression meta‐learner from the out‐of‐fold predictions. These coefficients are conditional regression parameters rather than normalized averaging weights and are not constrained to sum to one.

The inverse regularization‐strength parameter C of the meta‐learner was optimized over 13 logarithmically spaced values from 10^−3^ to 10^3^, with the value producing the highest mean internal AUC retained. For prediction on the held‐out spatial fold, the base learners were refitted using the complete outer training dataset and their selected hyperparameters. Their predicted probabilities were then supplied to the optimized meta‐learner to obtain the ensemble prediction according to Equation (1). Thus, the final stacking probability was not obtained through arithmetic averaging or predefined equal or AUC‐based weights. Instead, the logistic‐regression meta‐learner estimated coefficients and an intercept from the three base‐model probabilities and applied a logistic transformation.

Because hyperparameter tuning was conducted independently within each outer training dataset, the selected values could differ among folds. The fold‐specific optimal hyperparameters for the three base learners and the stacking meta‐learner are reported in Table S1.

To facilitate comparison of environmental effects across algorithms, variable importance was quantified using the same permutation‐based procedure for the RF, MaxEnt, GAM, and ensemble models. For each model fitted within an outer spatial fold, the baseline AUC was calculated using the corresponding fold‐training data. Each predictor was then randomly permuted 20 times while all other predictors were retained unchanged, and its importance was quantified as the mean decrease in AUC relative to the baseline value. Negative mean AUC decreases were set to zero, and the resulting importance values were normalized to sum to 100% within each model and fold. The final values were summarized as the mean ± standard deviation across the five outer spatial folds.

Model performance was evaluated separately on the five held‐out spatial folds and summarized as the mean and standard deviation. For final habitat‐suitability mapping, each fold‐specific model was used to predict probabilities of presence across the study area. For each model type, the five resulting probability rasters were averaged cell by cell to obtain the final habitat‐suitability prediction.

The analytical workflow was implemented using custom Python and R scripts. Python was used for raster value extraction, environmental variable screening, RF and GAM modeling, stacking ensemble construction, model evaluation, variable importance analysis, response‐curve calculation, and spatial raster prediction. MaxEnt was implemented by calling the R package dismo through the rpy2 interface. ArcGIS was used for suitability reclassification, area statistics, and centroid analysis. Details of the computational environment, hardware specifications, runtime, and software and package versions used in this study are provided in Table S2.

#### 2.4.2 Model Evaluation

To comprehensively evaluate model performance, a multidimensional evaluation framework was established to assess the overall discrimination ability, threshold‐dependent classification performance, and recognition accuracy under high‐specificity conditions. In terms of classification accuracy, the area under the receiver operating characteristic curve (AUC), true skill statistic (TSS), and partial AUC were used. AUC measures the overall ability of a model to distinguish species presence from absence by calculating the area under the ROC curve. Its value ranges from 0 to 1, where 0.5 indicates random prediction and 1 indicates perfect discrimination. As a threshold‐independent metric, AUC can effectively evaluate the overall accuracy of predicted species distribution probabilities and is one of the most commonly used evaluation indicators in SDM studies (Melo 2013; Zhu et al. 2017).

TSS integrates sensitivity and specificity and evaluates binary classification performance under a specific threshold, reflecting the model's ability to correctly identify both presence and absence records. In this study, TSS was calculated following the threshold‐dependent evaluation criterion proposed by Allouche et al. (2006). Its value ranges from −1 to 1, where 0 indicates performance no better than random prediction and 1 indicates perfect classification. Compared with metrics based on a single dimension, TSS provides a more comprehensive assessment of model performance under threshold‐based classification conditions.

In addition, conventional AUC has several limitations in the evaluation of species distribution models because it summarizes discrimination across the entire ROC curve and assigns equal importance to all false‐positive‐rate regions. Consequently, a high full AUC may obscure differences among models within the restricted high‐specificity region, which may be particularly important when false‐positive predictions need to be minimized. Partial AUC addresses this limitation by evaluating only a predefined portion of the ROC curve (Peterson et al. 2008; Cobos et al. 2019; Peterson et al. 2024; Richardson et al. 2024).

In this study, partial AUC was calculated over the false‐positive‐rate range of 0–0.1. The upper FPR limit of 0.1 was selected a priori to focus model evaluation on the high‐specificity portion of the ROC curve. This threshold corresponds to a specificity of at least 0.90, allowing no more than 10% of pseudo‐absence records to be incorrectly classified as presences, and therefore provides a stringent but interpretable criterion for evaluating discrimination when false‐positive predictions are constrained. The area under this restricted portion of the ROC curve was divided by the width of the selected false‐positive‐rate interval, yielding a width‐normalized partial AUC. The resulting value represents the mean true‐positive rate across the FPR range of 0–0.1. This measure differs from the McClish‐standardized partial AUC, in which random performance is rescaled to 0.5. Under the scaling used in this study, the theoretical maximum is 1.0, whereas the expected value for a random classifier is 0.05. Higher values therefore indicate that a model can identify a larger proportion of presence records while maintaining a false‐positive rate below 0.1. For example, a width‐normalized partial AUC of 0.50 means that, on average, the model correctly identifies about 50% of presence records while the false‐positive rate is constrained between 0 and 0.10.

These three metrics complement each other and evaluate model performance from three perspectives: overall ranking ability, threshold‐dependent classification accuracy, and high‐specificity discrimination accuracy. Together, they help overcome the limitations of a single metric and provide a more robust statistical basis for model selection and reliability analysis.

To formally determine whether the observed performance differences among models were statistically meaningful, paired‐sample *t*‐tests were conducted because all models were evaluated on the same five held‐out spatial folds. Specifically, the fold‐level AUC, TSS, and partial AUC values of the stacking ensemble were compared with the corresponding values of each base model. Wilcoxon signed‐rank tests were additionally performed as a nonparametric robustness check. For each test type, the nine *p* values arising from three performance metrics × three base‐model comparisons were treated as one family and adjusted separately using the Holm step‐down procedure. The nine raw *p* values were ranked from smallest to largest and sequentially compared with significance thresholds of α/9, α/8, …, α, thereby controlling the family‐wise error rate at α = 0.05.

#### 2.4.3 Potential Suitable Habitat Analysis

ArcGIS was used for geospatial analysis and visualization of the model prediction results. First, for the RF, MaxEnt, GAM, and ensemble models, the five fold‐specific species distribution probability rasters generated through outer spatial cross‐validation were averaged cell by cell to obtain the final predicted potential suitable‐habitat distributions. These raster outputs were then imported into GIS and overlaid with the vector maps of China's national and provincial administrative boundaries for spatial visualization. Subsequently, the reclassification tool in the spatial analysis module was used to classify the raster data according to habitat‐suitability probability thresholds for *P. tabuliformis* . Four suitability classes were defined: highly suitable habitat (≥ 0.6), moderately suitable habitat (0.4–0.6), lowly suitable habitat (0.2–0.4), and unsuitable habitat (< 0.2) (Qin et al. 2017; Wang et al. 2021). These class boundaries were adopted as operational thresholds to facilitate map interpretation, spatial comparison, and area estimation, rather than as empirically derived ecological thresholds for *P. tabuliformis* . A species‐optimized threshold was not used because such criteria generally define a single binary suitable–unsuitable cutoff, whereas the present analysis required three thresholds to divide habitat suitability into four classes consistently across models. To assess the sensitivity of habitat‐area estimates to threshold selection, two alternative classification schemes were additionally evaluated. In addition to the baseline class boundaries of 0.20, 0.40, and 0.60, all thresholds were shifted downward by 0.05 (0.15, 0.35, and 0.55) and upward by 0.05 (0.25, 0.45, and 0.65). The areas of each suitability class were recalculated for RF, MaxEnt, GAM, and the ensemble model under all three schemes, and changes in total and highly suitable habitat areas and their cross‐model rankings were compared. The area of each suitability class was calculated by summing the actual areas of the corresponding raster cells. Uncertainty in the estimated habitat areas was quantified using 1000 paired nonparametric fold‐bootstrap resamples, and the 2.5th and 97.5th percentiles of the bootstrap estimates were used to derive 95% confidence intervals. To quantify spatial overlap among model predictions, pairwise Jaccard similarity indices were calculated for RF, MaxEnt, GAM, and the ensemble model using the final five‐fold mean probability rasters. The analysis was conducted separately for total suitable habitat (suitability ≥ 0.2) and highly suitable habitat (suitability ≥ 0.6). For each pair of models, the Jaccard index was calculated as the intersection area divided by the union area. In addition, the area jointly classified as suitable by all four models was calculated, and its proportion relative to the union area classified as suitable by at least one model was used to characterize overall cross‐model spatial agreement.

To further compare spatial differences among model predictions, suitability‐weighted centroids were calculated for the RF, MaxEnt, GAM, and ensemble models using their five‐fold mean probability rasters, by averaging raster‐cell coordinates in an equal‐area projection with predicted suitability probability and cell area as weights. The resulting centroid represents the geographic center of predicted habitat suitability rather than the actual center of observed populations or the realized species distribution. Centroid uncertainty was quantified using 1000 paired fold‐bootstrap resamples and represented by 95% confidence ellipses. The direction and distance of each base‐model centroid relative to the ensemble centroid were also calculated, with 95% bootstrap confidence intervals used to characterize uncertainty in displacement distance.

## 3 Results

### 3.1 Environmental Factor Analysis

In this study, the relative importance of environmental variables in the RF, MaxEnt, GAM, and ensemble models was evaluated using permutation importance (Table 2). Although the four models differed in their weighting of individual predictors, mean temperature of the driest quarter (BIO9) ranked first in all models, with permutation importance values of 73.31% ± 8.15% in RF, 32.42% ± 9.26% in MaxEnt, 38.53% ± 9.92% in GAM, and 62.41% ± 12.20% in the ensemble model. The particularly high values in RF and the ensemble model indicate that their predictions were strongly dependent on mean temperature of the driest quarter, consistently highlighting dry‐season thermal conditions as the dominant environmental constraint on the current potential distribution of *P. tabuliformis* .

**TABLE 2:** Relative permutation importance of environmental variables in the RF, MaxEnt, GAM, and ensemble models.

Environmental variable	RF (%)	MaxEnt (%)	GAM (%)	Ensemble (%)
BIO9	73.31 ± 8.15	32.42 ± 9.26	38.53 ± 9.92	62.41 ± 12.20
BIO18	14.45 ± 5.89	5.27 ± 1.59	23.55 ± 6.01	11.82 ± 4.25
DEM	6.13 ± 3.71	23.51 ± 4.05	24.01 ± 11.00	15.00 ± 8.55
AWC	3.04 ± 1.73	0.48 ± 0.43	5.54 ± 3.15	2.48 ± 1.05
LBGC	1.32 ± 1.10	20.11 ± 7.44	2.92 ± 1.17	3.71 ± 2.55
snow_05	0.27 ± 0.46	4.65 ± 5.45	0.08 ± 0.04	0.54 ± 0.72
BIO19	0.43 ± 0.63	2.83 ± 2.29	2.06 ± 1.53	1.69 ± 1.48
Upa	0.74 ± 0.41	4.86 ± 1.83	1.39 ± 0.61	1.24 ± 0.35
Slope	0.11 ± 0.11	0.21 ± 0.06	0.72 ± 0.50	0.25 ± 0.09
Aspect	0.12 ± 0.15	0.55 ± 0.26	0.90 ± 0.29	0.43 ± 0.17
Hand	0.08 ± 0.09	5.11 ± 1.58	0.30 ± 0.11	0.45 ± 0.13

*Note:* Values represent permutation importance calculated as the decrease in AUC after randomly permuting each predictor 20 times. Negative mean AUC decreases were set to zero, and the resulting importance values were normalized to sum to 100% within each model and fold. Values are presented as the mean ± SD across the five outer spatial folds. The MaxEnt values were calculated using the same external permutation procedure as those for the other models and do not represent native MaxEnt percent contribution or jackknife results.

The remaining importance patterns differed among algorithms. In GAM, importance was distributed relatively evenly among BIO9, DEM, and precipitation of the warmest quarter (BIO18), with values of 38.53% ± 9.92%, 24.01% ± 11.00%, and 23.55% ± 6.01%, respectively, indicating that this model captured the combined effects of thermal, topographic, and growing‐season moisture gradients. MaxEnt also ranked BIO9 first, while DEM and long‐term bare ground cover (LBGC) showed relatively high permutation importance, with values of 23.51% ± 4.05% and 20.11% ± 7.44%, respectively. This pattern indicates that topographic and land‐surface background conditions were also influential in the MaxEnt predictions. In RF, BIO18 was the second most important predictor, with a permutation importance of 14.45% ± 5.89%, and it also showed appreciable importance in the ensemble model (11.82% ± 4.25%). By contrast, AWC, BIO19, Upa, slope, aspect, HAND, and Snow_05 generally had lower importance across models. These contrasting importance profiles were consistent with differences in model structure, with RF and the ensemble showing more concentrated predictor dependence, whereas GAM and MaxEnt distributed predictive importance more broadly across multiple variables. Overall, despite algorithm‐specific differences in variable weighting, the four models consistently identified BIO9 as the dominant predictor, while DEM and BIO18 also emerged as important environmental variables across multiple models.

**FIGURE 3:**
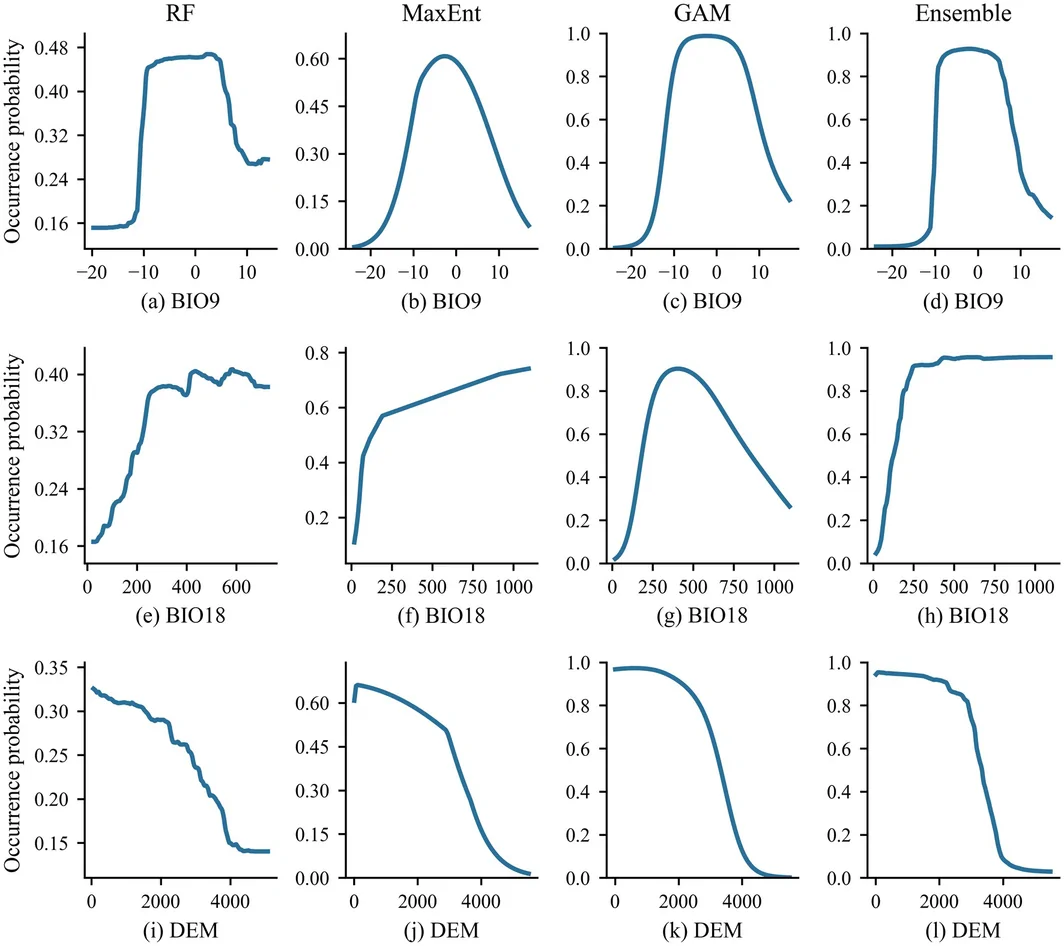
Partial dependence plots showing the responses of predicted occurrence probability to BIO9, BIO18, and DEM across the four models: (a) RF: BIO9; (b) MaxEnt: BIO9; (c) GAM: BIO9; (d) Ensemble: BIO9; (e) RF: BIO18; (f) MaxEnt: BIO18; (g) GAM: BIO18; (h) Ensemble: BIO18; (i) RF: DEM; (j) MaxEnt: DEM; (k) GAM: DEM; and (l) Ensemble: DEM. BIO9 represents mean temperature of the driest quarter, BIO18 represents precipitation of the warmest quarter, and DEM represents elevation.

The partial dependence plots further revealed both common and model‐specific responses of the predicted occurrence probability of *P. tabuliformis* to the three key environmental gradients (Figure 3). For BIO9, all four models predicted low occurrence probabilities under extremely cold conditions, followed by a marked increase as the mean temperature of the driest quarter approached approximately −10°C. RF, GAM, and the ensemble model showed relatively high and stable probabilities between approximately −10°C and 5°C, followed by a decline at higher temperatures, whereas MaxEnt exhibited a smoother unimodal response with a peak near the middle of this range. These patterns indicate that the predicted suitability of *P. tabuliformis* was highest under cool, but not extremely cold, dry‐season conditions.

The response to BIO18 was less consistent among models. All four models indicated relatively low occurrence probabilities under low warm‐season precipitation and a pronounced increase as precipitation increased to approximately 200–300 mm. Beyond this range, RF and the ensemble model generally approached a plateau; MaxEnt showed a continued gradual increase, whereas GAM reached its highest probability at approximately 300–500 mm and subsequently declined. Thus, the models consistently indicated a constraint associated with insufficient warm‐season precipitation but differed in their responses under moderate to high precipitation conditions.

DEM showed the most consistent response across models. Predicted occurrence probability generally declined with increasing elevation. Suitability remained relatively high at low to mid elevations, particularly below approximately 2000–2500 m, and decreased markedly above approximately 3000 m. This decline was relatively gradual in RF but became considerably steeper in MaxEnt, GAM, and the ensemble model between approximately 3000 and 4000 m, indicating reduced suitability at high elevations.

Overall, the response patterns were most consistent for DEM and for the low‐temperature portion of BIO9, whereas BIO18 exhibited greater algorithm‐specific variation at moderate to high values. Together with the permutation‐importance results, these response curves consistently identified BIO9 as the dominant environmental predictor, while DEM and BIO18 represented additional topographic and growing‐season moisture constraints on the current potential distribution of *P. tabuliformis* . Taken together, these findings generally support the first hypothesis that seasonal hydrothermal conditions and topography, particularly mean temperature of the driest quarter (BIO9), precipitation of the warmest quarter (BIO18), and elevation, are major predictors of habitat suitability for *P. tabuliformis* .

### 3.2 Model Accuracy Comparison

**FIGURE 4:**
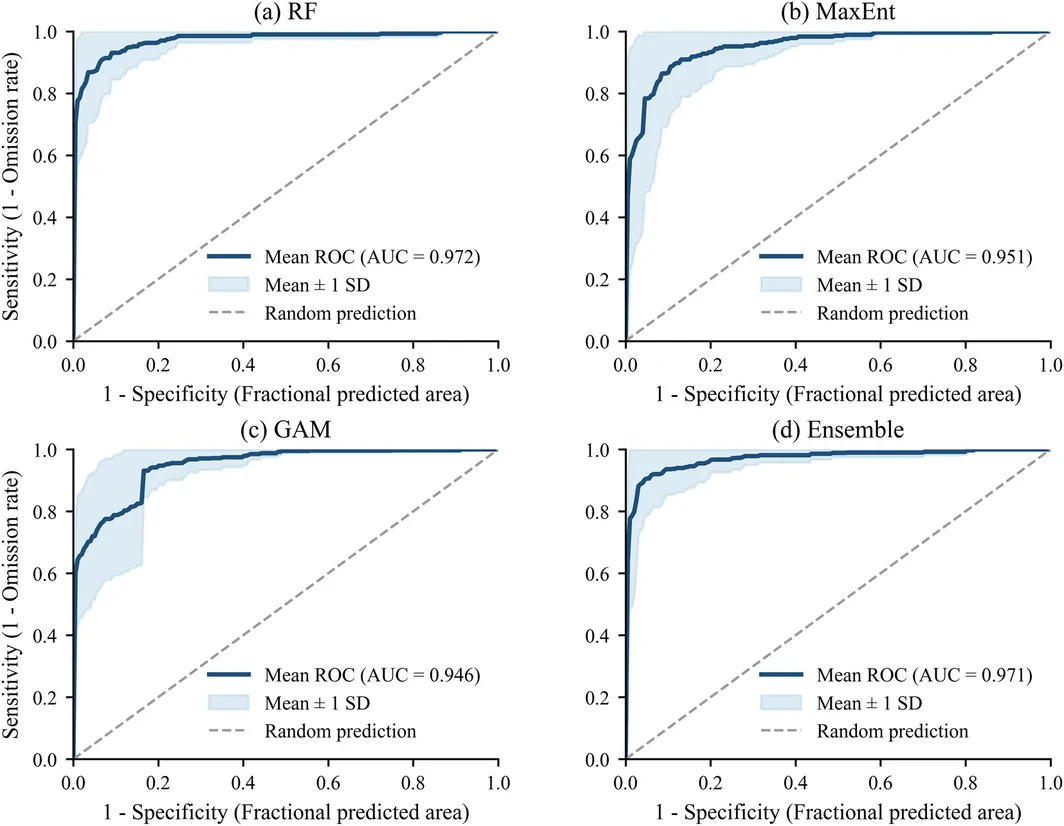
Receiver operating characteristic curves of the four species distribution models: (a) RF; (b) MaxEnt; (c) GAM; and (d) Ensemble learning model.

In this study, model performance was evaluated using predictions from the five held‐out spatial folds. For each model, receiver operating characteristic curves were generated from the fold‐specific test predictions, and the evaluation metrics were summarized as the mean ± standard deviation across the five folds (Figure 4). The ROC curves of all four models were generally above the random prediction baseline, indicating that all models could effectively distinguish presence records from pseudo‐absence records under spatially independent validation. However, differences among AUC, TSS, and partial AUC showed that strong overall discrimination did not necessarily correspond to equally strong threshold‐dependent or high‐specificity performance.

RF achieved the highest mean AUC among the four models, with a value of 0.973 ± 0.031, indicating the strongest overall discrimination across the full range of classification thresholds (Table 3). The ensemble model showed nearly identical performance, with an AUC of 0.972 ± 0.034, whereas MaxEnt and GAM obtained slightly lower AUC values of 0.952 ± 0.054 and 0.947 ± 0.037, respectively. These results indicate that the stacking ensemble maintained overall discrimination comparable to that of the best‐performing individual model but did not further improve mean AUC. The relatively larger AUC variation of MaxEnt also suggests that its discrimination performance was more sensitive to differences among the held‐out spatial folds. Consistent with the small numerical difference between RF and the ensemble, the paired comparison across the five spatial folds showed no statistically significant difference in AUC between the two models (paired‐sample *t*‐test, *p* = 0.785; Wilcoxon signed‐rank test, *p* = 1.000).

**TABLE 3:** Comparison of model performance.

Model	TSS	Width‐normalized partial AUC (0–0.1)	AUC
RF	0.726 ± 0.140	0.574 ± 0.245	0.973 ± 0.031
Maxent	0.565 ± 0.353	0.383 ± 0.120	0.952 ± 0.054
GAM	0.645 ± 0.134	0.392 ± 0.142	0.947 ± 0.037
Ensemble Learning	0.714 ± 0.157	0.490 ± 0.055	0.972 ± 0.034

*Note:* Values are presented as mean ± SD across the five held‐out spatial folds. TSS was calculated using a probability threshold of 0.5. The width‐normalized partial AUC was calculated over the false‐positive‐rate range of 0–0.1 by dividing the restricted ROC area by the width of this interval; it therefore represents the mean true‐positive rate within this high‐specificity region. Under this scaling, the expected value for a random classifier is 0.05 and the theoretical maximum is 1.0. Higher values indicate better predictive performance.

Threshold‐dependent classification performance showed a broadly similar pattern. TSS reflects the balance between sensitivity and specificity at the selected classification threshold and therefore evaluates the ability of a model to correctly identify both presence and pseudo‐absence records. RF obtained the highest mean TSS value of 0.726 ± 0.140, indicating the strongest average threshold‐dependent classification performance. The ensemble model followed closely, with a TSS of 0.714 ± 0.157, whereas GAM and MaxEnt showed lower values of 0.645 ± 0.134 and 0.565 ± 0.353, respectively. The particularly large standard deviation of MaxEnt indicates substantial variation in its threshold‐based classification performance among the spatial folds. Although the ensemble model achieved a mean TSS close to that of RF, its slightly larger standard deviation did not indicate improved fold‐to‐fold stability for this metric. The difference between the ensemble and RF was also not statistically significant for TSS (paired‐sample *t*‐test, *p* = 0.755; Wilcoxon signed‐rank test, *p* = 1.000).

Model differences became more pronounced for partial AUC within the false‐positive rate range of 0–0.1, which evaluates discrimination under high‐specificity conditions while restricting the proportion of pseudo‐absence records incorrectly identified as suitable. RF achieved the highest mean partial AUC, with a value of 0.574 ± 0.245, indicating the strongest average discrimination within this restricted ROC region. However, its relatively large standard deviation revealed considerable variation among spatial folds. The ensemble model had a lower mean partial AUC of 0.490 ± 0.055 but showed the smallest variation, suggesting more consistent, although not stronger, performance under high‐specificity conditions. GAM and MaxEnt obtained lower mean partial AUC values of 0.392 ± 0.142 and 0.383 ± 0.120, respectively. These results indicate that model discrimination became more challenging when false‐positive predictions were strictly controlled and that partial AUC revealed spatial variation that was less apparent from the high overall AUC values. Nevertheless, the partial AUC difference between the ensemble and RF was not statistically significant (paired‐sample *t*‐test, *p* = 0.486; Wilcoxon signed‐rank test, *p* = 0.625).

Overall, RF achieved the highest mean values for AUC, TSS, and partial AUC, whereas the ensemble model remained very close to RF in both AUC and TSS but did not outperform it in mean predictive accuracy. Across the nine planned comparisons involving three evaluation metrics and three base models, none of the differences remained statistically significant after Holm correction (Table S3). The paired‐sample *t*‐tests and Wilcoxon signed‐rank tests therefore led to the same overall conclusion. The lower variation of the ensemble model was mainly observed for partial AUC and should therefore be interpreted as greater consistency within the restricted high‐specificity region rather than uniformly greater robustness across all evaluation criteria. These results support our hypothesis that the stacking ensemble would not necessarily outperform the individual models across all evaluation metrics under spatially independent validation.

### 3.3 Potential Suitable Habitat Distribution of *Pinus tabuliformis*

**FIGURE 5:**
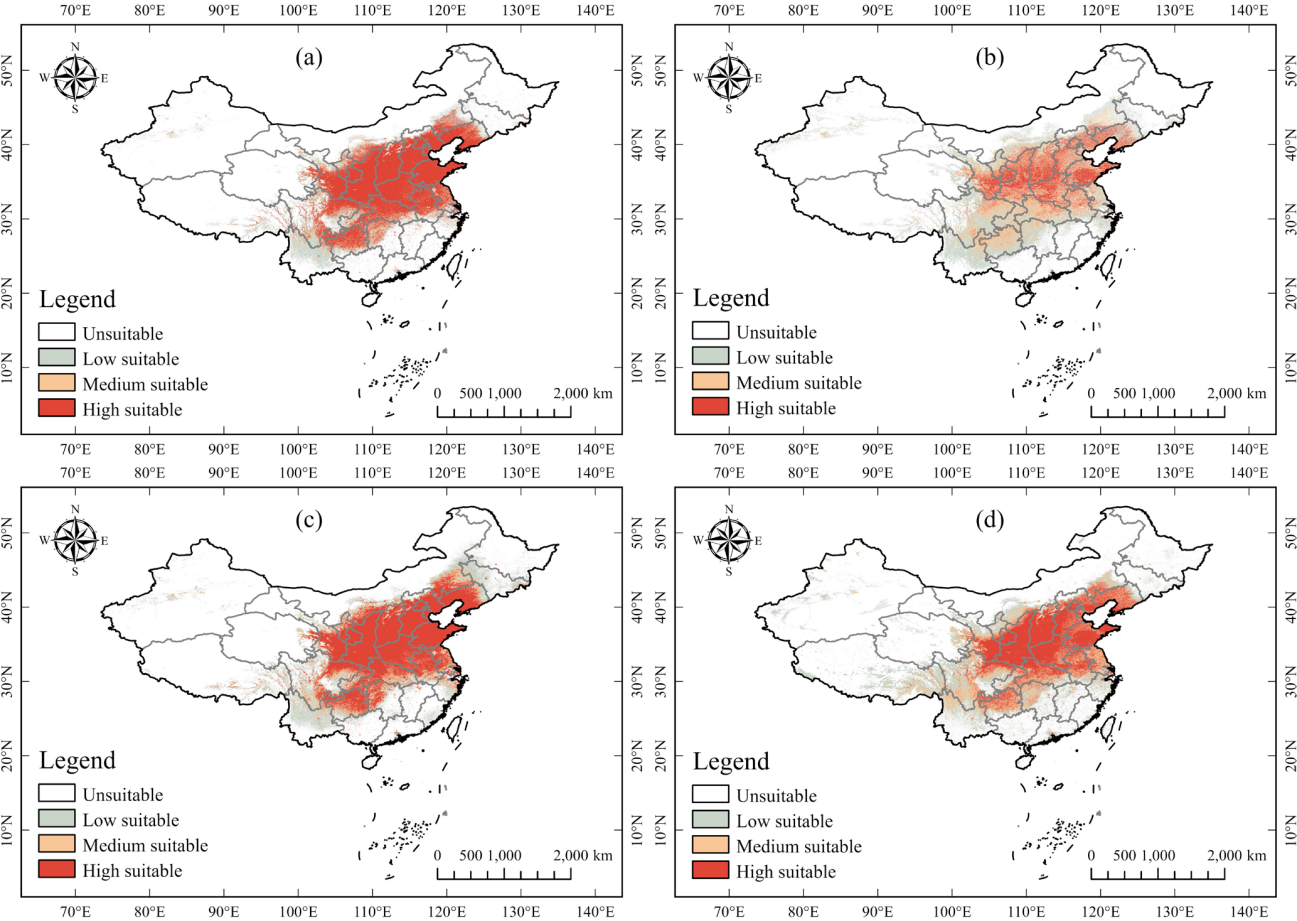
Predicted potential suitable habitats of *Pinus tabuliformis* under different models: (a) ensemble learning model; (b) MaxEnt model; (c) GAM model; and (d) RF model.

The predicted potential suitable habitats of *P. tabuliformis* under current environmental conditions are shown in Figure 5. All four models, including MaxEnt, RF, GAM, and the ensemble model, indicated that highly suitable habitats were mainly distributed in low‐ to mid‐elevation mountainous and hilly areas of northern China, including southeastern Hebei, southern Shanxi, central Shaanxi, and southern Liaoning. Moderately suitable habitats were more widely distributed, mainly surrounding the highly suitable areas and extending into parts of the Loess Plateau. Lowly suitable habitats were mainly distributed in northeastern China and southern Inner Mongolia. The four models showed broadly similar regional patterns but differed in their degree of spatial overlap and in the allocation of area among suitability classes. For total suitable habitat (suitability ≥ 0.2), pairwise Jaccard indices ranged from 0.651 to 0.789, with the greatest overlap between GAM and the ensemble model and the lowest between RF and MaxEnt. The area jointly classified as suitable by all four models was 23.08 × 10^5^ km^2^, accounting for 56.7% of the four‐model suitable‐area union. Differences were more pronounced for highly suitable habitat (suitability ≥ 0.6), for which pairwise Jaccard indices ranged from 0.321 to 0.783. The four‐model common highly suitable area was 6.05 × 10^5^ km^2^, accounting for 28.3% of the corresponding union (Table S4). Overall, this spatial pattern supports the third hypothesis that highly suitable habitats are concentrated mainly in low‐ to mid‐elevation mountainous and hilly regions of northern and north‐central China, particularly across the Loess Plateau and adjacent areas. In addition, the Qinling Mountains formed an important southern marginal zone of potential suitability.

### 3.4 Centroid and Area Analysis of Suitable Habitats for *Pinus tabuliformis*

**FIGURE 6:**
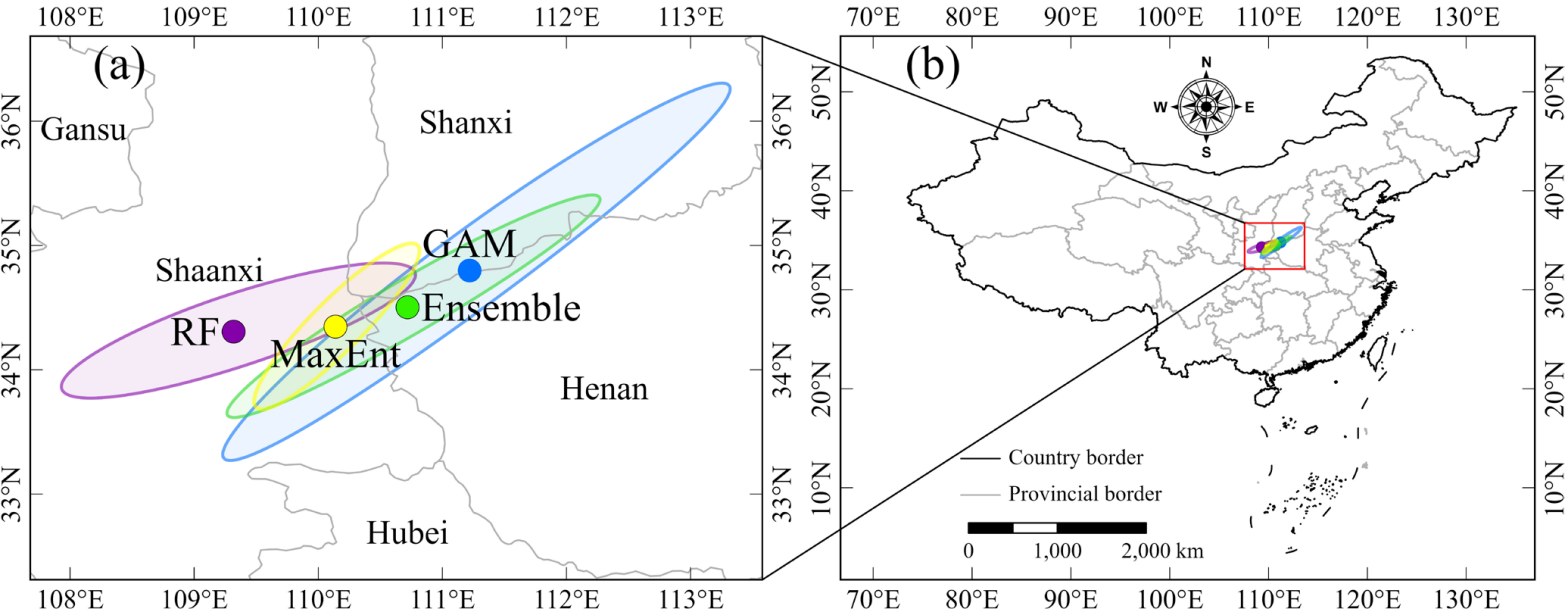
Spatial locations and uncertainty of the suitability‐weighted centroids predicted by the RF, MaxEnt, GAM, and ensemble models. (a) Colored points represent the suitability‐weighted centroids calculated from the five‐fold mean probability rasters, and the corresponding semi‐transparent ellipses represent the 95% bootstrap standard deviational regions derived from 1000 paired fold‐based bootstrap resamples. (b) Location of the enlarged area within China. The ellipses reflect variation among the five outer spatial folds and do not represent the geographic extent of suitable habitat.

The current centroid of suitable habitats for *P. tabuliformis* was located in central China (Figure 6), with the centroid predicted by the ensemble model at approximately 34.50° N and 110.72° E. Based on 1000 paired fold‐based bootstrap resamples, the coordinate‐specific 95% confidence intervals of the ensemble centroid were 33.86°–35.15° N for latitude and 109.61°–111.64° E for longitude, indicating a moderate level of spatial uncertainty associated with variation among the five outer spatial folds. The 95% bootstrap standard deviational ellipse was smallest for MaxEnt, indicating the greatest spatial concentration of its centroid estimates, whereas GAM showed the largest ellipse and thus the greatest spatial uncertainty. The RF ellipse was relatively elongated, suggesting pronounced directional variation in centroid location, while the ensemble model exhibited an intermediate level of spatial uncertainty.

Using the centroid of the ensemble model as a reference, the RF centroid was displaced by 128.54 km to the southwest (95% CI: 105.49–155.02 km), the MaxEnt centroid by 55.32 km to the southwest (95% CI: 9.01–107.09 km), and the GAM centroid by 56.03 km to the northeast (95% CI: 12.15–118.19 km). Although MaxEnt had the smallest uncertainty ellipse for centroid location, the RF‐to‐ensemble displacement had the narrowest 95% confidence interval, indicating lower uncertainty in the estimated displacement distance. Overall, the three single models differed in both the direction and magnitude of centroid displacement, although all predicted centroids remained within central China. The ensemble centroid was located between the more southwesterly RF and MaxEnt estimates and the more northeasterly GAM estimate, reflecting the integration of spatial predictions from the base models. This result provides a synthesized estimate of the geographic center of the current potentially suitable habitat for *P. tabuliformis* .

**FIGURE 7:**
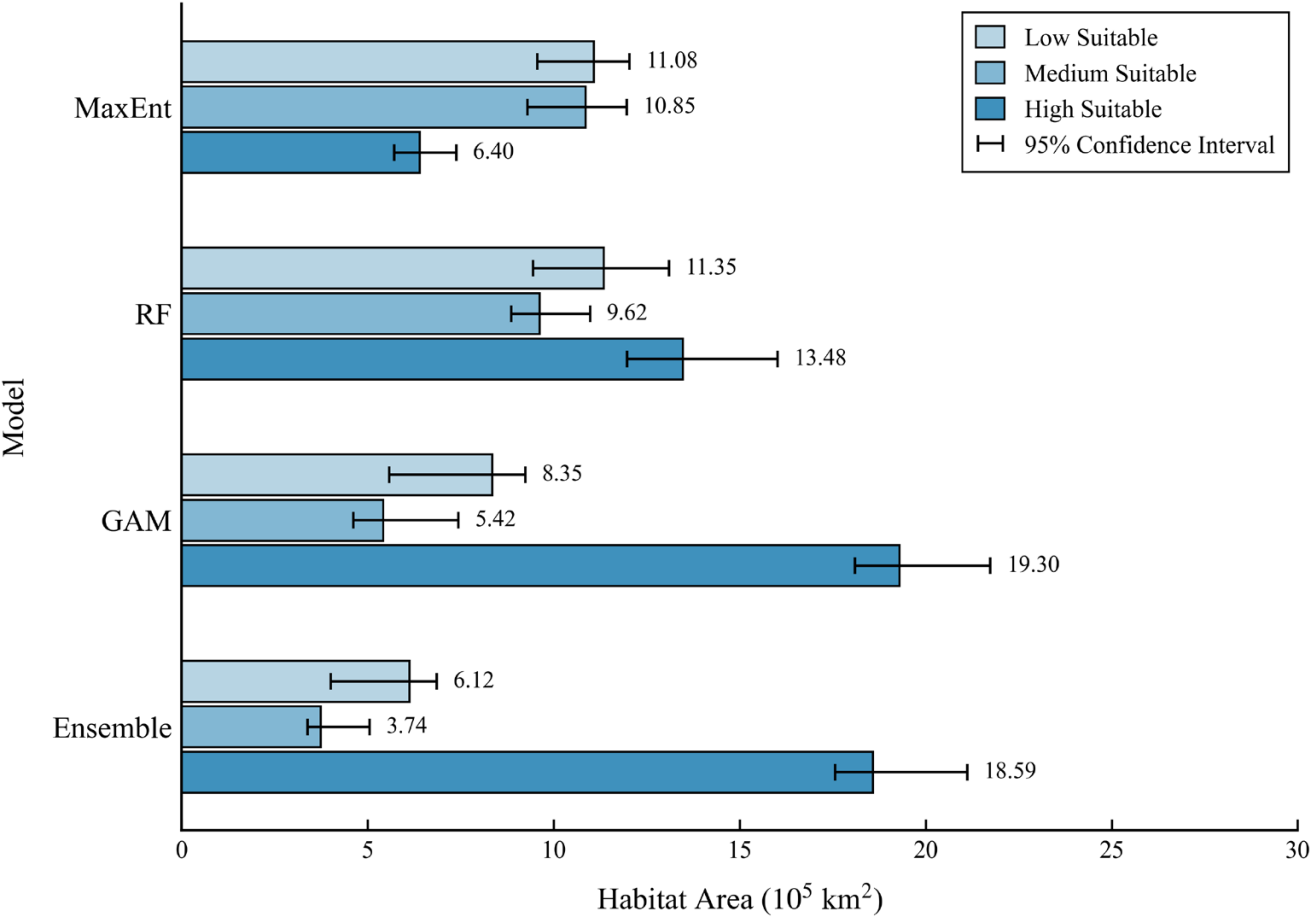
Predicted areas of low suitability, moderate suitability, and high suitability habitats for *Pinus tabuliformis* under the MaxEnt, RF, GAM, and ensemble models. Bars represent the estimated habitat areas, numbers indicate the corresponding area values, and error bars denote the 95% confidence intervals derived from 1000 paired fold‐based bootstrap resamples.

The total area classified as suitable for *P. tabuliformis* differed among models (Figure 7). The RF model predicted the largest total suitable area, reaching 34.44 × 10^5^ km^2^ (95% CI: 30.76–39.99 × 10^5^ km^2^), followed by the GAM model at 33.07 × 10^5^ km^2^ (95% CI: 28.69–37.24 × 10^5^ km^2^). The total suitable areas predicted by the ensemble and MaxEnt models were 28.45 × 10^5^ km^2^ (95% CI: 25.23–31.42 × 10^5^ km^2^) and 28.34 × 10^5^ km^2^ (95% CI: 27.24–28.82 × 10^5^ km^2^), respectively. Relative to the RF estimate, the total suitable areas predicted by GAM, the ensemble model, and MaxEnt were lower by approximately 4.0%, 17.4%, and 17.7%, respectively. However, the distribution among suitability classes also differed among models. GAM and the ensemble model predicted the largest highly suitable areas, at 19.30 × 10^5^ km^2^ and 18.59 × 10^5^ km^2^, respectively, whereas RF and MaxEnt predicted 13.48 × 10^5^ km^2^ and 6.40 × 10^5^ km^2^. These results indicate that model differences involved not only the total extent of suitable habitat but also the allocation of area among low, moderate, and high suitability classes. The narrower confidence interval for MaxEnt indicated relatively low variation in the total suitable‐area estimate among the five outer spatial folds, whereas RF and GAM showed greater uncertainty.

To examine whether these area differences depended on the selected classification thresholds, a threshold‐sensitivity analysis was conducted using two alternative threshold schemes (Table S5). The principal cross‐model patterns were generally maintained under moderate changes in the classification thresholds. RF consistently predicted the largest total suitable area and GAM the second largest under all three threshold schemes. The relative ordering of MaxEnt and the ensemble changed under the lower‐threshold scheme, indicating some sensitivity in their total suitable‐area estimates. In contrast, the ranking of highly suitable habitat remained unchanged across all schemes (GAM > Ensemble > RF > MaxEnt). Relative to the baseline scheme, the ensemble showed comparatively small changes in highly suitable area (−5.25% to +5.01%), whereas MaxEnt showed greater sensitivity (−37.22% to +37.73%).

## 4 Discussion

### 4.1 Predictive Performance and Role of the Stacking Ensemble

Under spatial block cross‐validation, a key finding was that the stacking ensemble did not outperform RF, despite both models showing strong overall discrimination. RF achieved slightly higher mean AUC, TSS, and width‐normalized partial AUC values, whereas the ensemble showed AUC and TSS performance close to RF. Moreover, none of the pairwise differences between the ensemble and the three base models remained statistically significant after Holm correction. These results support the second hypothesis that the stacking ensemble would not necessarily outperform individual models across all evaluation metrics under spatially independent validation. Previous comparisons have similarly shown that ensemble species distribution models do not invariably exceed the best‐performing individual model because their relative performance depends on the quality and complementarity of the base learners, the method used to combine them, and the validation design (Marmion et al. 2009; Hao et al. 2019, 2020; Oeser et al. 2024).

The width‐normalized partial AUC provided a complementary assessment of model performance when the false‐positive rate was restricted to 0–0.1. Under the scaling used in this study, the random‐classifier reference is 0.05 rather than 0.5, and all four models performed well above this reference. RF achieved the highest mean partial AUC but also showed the greatest variation among spatial folds, whereas the ensemble had a lower mean value and markedly smaller fold‐to‐fold variation. Therefore, under high‐specificity conditions, RF showed higher mean sensitivity, whereas the ensemble produced more consistent results across spatial folds.

Differences among models were more evident in their spatial predictions than in their evaluation metrics, reflecting the distinct ways in which the algorithms represented species–environment relationships. RF predicted the largest total suitable area. Its tree‐based structure can accommodate complex nonlinear responses and interactions among environmental predictors, which may allow suitable environmental combinations to be identified across a relatively broad environmental space (Cutler et al. 2007). However, the larger predicted area cannot by itself be interpreted as overprediction, because independent occurrence records or field validation would be required to distinguish genuine additional suitable habitat from algorithm‐specific spatial extension.

MaxEnt and GAM showed different spatial patterns that were also consistent with their model structures. MaxEnt uses transformed environmental features and regularization to constrain model complexity, and in this study it produced a relatively restricted total suitable area and the smallest highly suitable area (Phillips and Dudík 2008; Elith et al. 2011). These results should not be attributed to superior performance under high‐specificity conditions because MaxEnt did not achieve the highest width‐normalized partial AUC. Rather, they indicate a comparatively restrained allocation of high suitability under the fitted feature and regularization settings. GAM, in contrast, represents species–environment relationships through additive smooth functions. This structure facilitates the estimation of continuous and interpretable nonlinear responses; however, under its default additive structure, its ability to represent complex higher‐order interactions and abrupt response boundaries is generally weaker than that of RF (Yee and Mitchell 1991; Wood 2017). The comparatively large total and highly suitable areas predicted by GAM may therefore be associated with its smooth representation of environmental gradients and transitions in habitat suitability.

The ensemble produced a smaller total suitable area than RF and GAM but retained a comparatively large highly suitable area, indicating that it did not simply contract the predictions of all base models. Its suitability‐weighted centroid was located between the more southwesterly RF and MaxEnt estimates and the more northeasterly GAM estimate. This intermediate position is consistent with the stacking framework integrating spatial information from the three base models. Rather than calculating a simple average, the logistic‐regression meta‐learner combined their out‐of‐fold probability predictions using relationships learned from the training data (Wolpert 1992; Breiman 1996). By learning how to combine predictions from structurally different algorithms, the stacking framework reduced reliance on any single model and generated a synthesized spatial estimate, although the intermediate centroid uncertainty showed that model integration did not necessarily produce the lowest spatial uncertainty. More generally, these differences demonstrate that models with similar discrimination statistics may still produce substantially different estimates of suitable area and niche boundaries (Syphard and Franklin 2009; Norberg et al. 2019). The spatial‐overlap analysis quantified this divergence. The four‐model intersection accounted for 56.7% of the union of total suitable habitat but only 28.3% of the union of highly suitable habitat, indicating that model agreement was stronger for the broad extent of suitable habitat than for the spatial delineation of highly suitable areas. Because these area comparisons may also depend on the selected classification thresholds, we further evaluated threshold sensitivity. Although the magnitude of predicted area differences varied with the selected thresholds, particularly for the highly suitable area predicted by MaxEnt, the cross‐model ranking of highly suitable area remained unchanged across the tested threshold schemes.

These findings also have practical implications for model choice. When the primary objective is to maximize mean predictive discrimination, RF may be preferred because it achieved the highest mean AUC, TSS, and width‐normalized partial AUC, although its performance was not statistically superior to the other models after Holm correction. GAM may be useful when smooth and interpretable species–environment relationships are a priority, whereas the stacking ensemble is appropriate when a synthesized spatial estimate that reduces reliance on any single algorithm is desired. For management applications, areas consistently identified as suitable across models can be regarded as higher‐confidence candidates, whereas areas of strong model disagreement should be prioritized for field validation.

### 4.2 Driving Mechanisms of Key Environmental Factors

The results (Table 2) showed that BIO9 (mean temperature of the driest quarter), DEM, and BIO18 (precipitation of the warmest quarter) constituted the principal environmental gradients associated with the current potential distribution of *P. tabuliformis* . However, the concentration and distribution of permutation importance differed markedly among algorithms. In RF, variable importance was strongly concentrated on BIO9, which accounted for 73.31% ± 8.15% of the normalized permutation importance, followed by BIO18 at 14.45% ± 5.89% and DEM at 6.13% ± 3.71%. The ensemble model showed a similarly concentrated pattern, with BIO9, DEM, and BIO18 accounting for 62.41% ± 12.20%, 15.00% ± 8.55%, and 11.82% ± 4.25%, respectively. By contrast, GAM distributed importance more evenly among BIO9 (38.53% ± 9.92%), DEM (24.01% ± 11.00%), and BIO18 (23.55% ± 6.01%). MaxEnt also exhibited a less concentrated importance profile than RF, with importance distributed across BIO9 (32.42% ± 9.26%), DEM (23.51% ± 4.05%), LBGC (20.11% ± 7.44%), and several secondary predictors, including HAND, Upa, BIO18, and Snow_05.

These differences in importance concentration are consistent with the distinct ways in which the algorithms represent species–environment relationships. In RF, recursive tree splitting can produce predictions that depend strongly on a predictor when that variable repeatedly provides informative partitions of the environmental space; this is consistent with the pronounced concentration of permutation importance on BIO9 (Breiman 2001; Cutler et al. 2007). In contrast, GAM represents predictor effects through additive smooth functions, allowing predictive information to be distributed among several continuous environmental gradients, as reflected by the more balanced importance of BIO9, DEM, and BIO18 (Yee and Mitchell 1991; Wood 2017). MaxEnt represents environmental relationships through multiple transformed features under regularization, which can distribute predictive information across several predictors and feature forms rather than concentrating it in a single variable (Phillips and Dudík 2008; Elith et al. 2011; Merow et al. 2013). The ensemble importance reflects the dependence of the entire stacking prediction on each environmental variable: predictors first influence the probabilities generated by the three base learners, which are then integrated by the logistic‐regression meta‐learner (Wolpert 1992; Breiman 1996). Thus, the high importance of BIO9 in the ensemble indicates that its predictive signal remained influential after integration of the three structurally different base models.

In addition, the importance values reported in this study were calculated using the same permutation‐based decrease in AUC for RF, MaxEnt, GAM, and the ensemble model, rather than using the native percent‐contribution statistic produced by MaxEnt. When several predictors contain complementary or partially overlapping environmental information, permuting one variable may cause only a moderate decrease in model performance because part of its predictive signal remains represented by other variables and features. The normalized permutation importance may therefore be distributed across a broader set of predictors. Accordingly, the more even importance profile of MaxEnt should not be interpreted as evidence that it identified weaker environmental effects. Instead, it reflects an algorithm‐specific way of representing the multivariate environmental niche. Cross‐model interpretation should therefore emphasize the consistency of variable rankings and response patterns rather than direct comparisons of absolute importance percentages (Smith and Santos 2020). Although model structure may partly explain the particularly concentrated BIO9 importance in RF and the ensemble, the dominance of BIO9 is unlikely to be solely algorithmic. BIO9 ranked first in all four structurally different models, including GAM and MaxEnt, where its normalized importance was less concentrated, and the four models showed broadly consistent response patterns to BIO9. This cross‐model consistency suggests that the exceptionally high values in RF and the ensemble reflect both algorithm‐specific concentration of predictive dependence and a genuine ecological signal associated with dry‐season thermal conditions. DEM and BIO18 also repeatedly emerged as important predictors across models, further supporting their identification as robust environmental constraints on the current potential distribution of *P. tabuliformis* .

**FIGURE 8:**
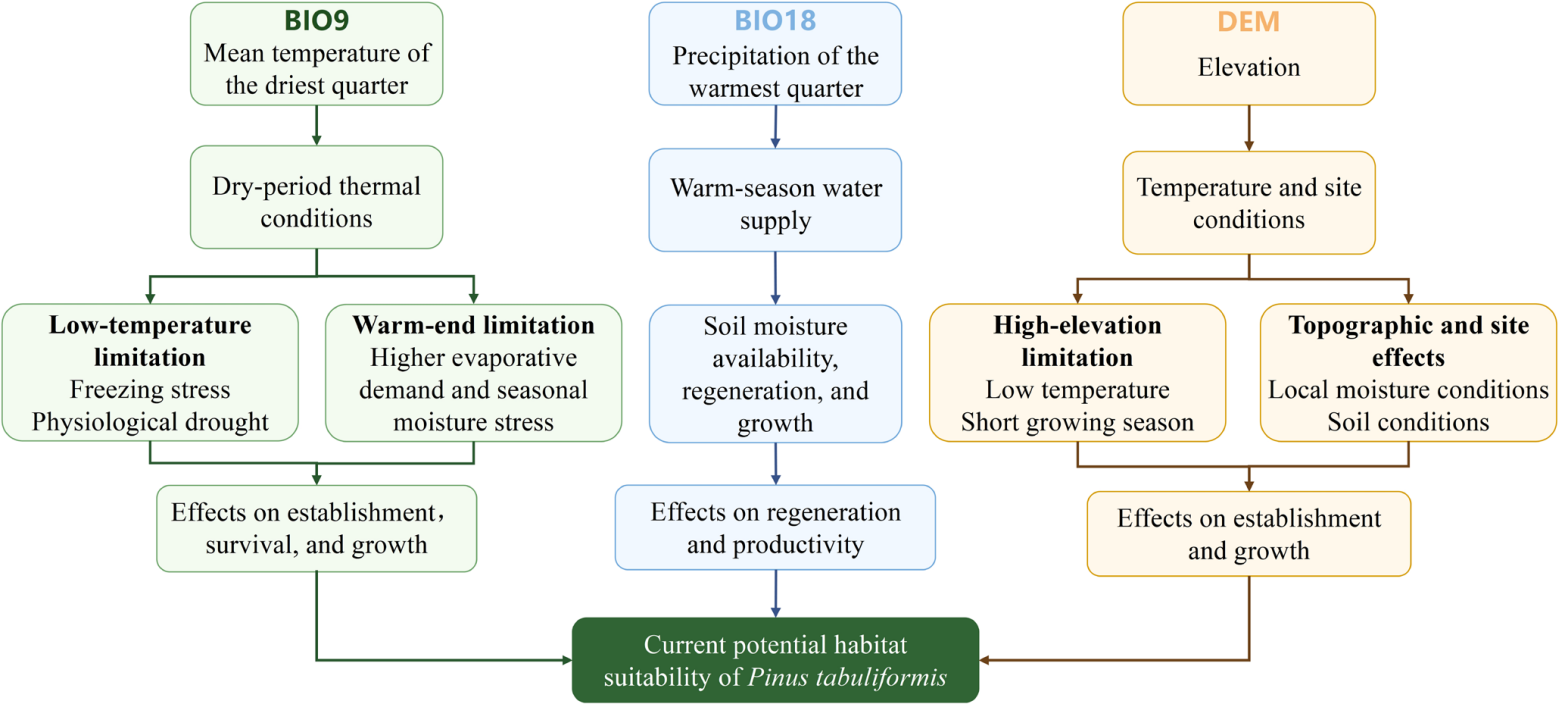
Conceptual framework linking the major environmental predictors to potential physiological and ecological mechanisms affecting the habitat suitability of *Pinus tabuliformis* . BIO9 represents mean temperature of the driest quarter, BIO18 represents precipitation of the warmest quarter, and DEM represents elevation. The pathways summarize mechanisms supported by the model response patterns and previous ecological studies.

Figure 8 conceptually summarizes the potential physiological and ecological pathways linking BIO9, DEM, and BIO18 to the habitat suitability of *P. tabuliformis* . Among these factors, BIO9 had clear ecophysiological significance in constraining the distribution of *P. tabuliformis* . *P. tabuliformis* is mainly distributed in warm‐temperate to temperate mountainous regions of China, and the driest quarter generally corresponds to winter and early spring. During this period, precipitation is limited and soil water availability is low, exposing plants simultaneously to low‐temperature stress and physiological drought. The response curves in this study showed that the suitable range of mean temperature of the driest quarter was −10°C to 5°C, indicating that thermal conditions during this period are directly related to overwintering survival, spring recovery, and subsequent growth performance of *P. tabuliformis* . When temperature falls below this range, the risks of freezing injury and physiological drought increase; when temperature exceeds this range, warmer winter conditions may weaken dormancy processes and intensify heat–moisture stress along the southern distribution margin (Qu et al. 2025; Zhang et al. 2026).

This temperature threshold also helps explain the northern and southern distribution boundaries of *P. tabuliformis* . In northern and high‐elevation regions, low temperature and insufficient accumulated heat reduce habitat suitability. At the southern margin, all four models showed declining suitability toward the warmer end of BIO9, although the shape and onset of this decline differed among algorithms. This consistent warm‐end response indicates that greater heat availability does not necessarily increase habitat suitability. The southern limit is more likely associated with temperature‐induced seasonal moisture stress than with direct heat injury alone. In the Qinling–Funiu warm‐temperate transition zone, higher temperatures during spring and the early growing season may increase evaporation and tree transpiration before the main monsoon rainfall becomes available, accelerating soil‐water depletion and inducing physiological drought. Tree‐ring studies from the Funiu Mountains and the southern slope of the central Qinling Mountains similarly identified high May temperatures and the resulting water stress as important limitations on the radial growth of *P. tabuliformis* (Peng, Peng, et al. 2025; Chen et al. 2025). Competition may further reinforce this climatic constraint locally. Because *P. tabuliformis* is light demanding, greater canopy closure and competition from broadleaved or more shade‐tolerant species may restrict seedling recruitment in productive southern forests; field observations have shown that its seedling density increases with understory light availability (Yang et al. 2023). However, because forest composition and competition were not explicitly represented in the present models, competition should be regarded as a plausible local reinforcing mechanism rather than a directly tested cause. Overall, the southern distribution limit is most plausibly associated with heat‐induced seasonal moisture stress, while competition and regeneration limitation may further modify the realized boundary at local scales.

DEM, as an important topographic factor, reflects the preference of *P. tabuliformis* for low‐ to mid‐elevation mountainous environments. Elevation comprehensively influences regional temperature, moisture availability, radiation, and soil conditions. The response curves showed a relatively consistent decline in predicted occurrence probability with increasing elevation. Suitability remained comparatively high at low to intermediate elevations, particularly below approximately 2000–2500 m, but decreased markedly above approximately 3000 m. The decline was gradual in RF but considerably steeper in MaxEnt, GAM, and the ensemble model between approximately 3000 and 4000 m. At high elevations, low temperatures, reduced accumulated heat, shortened growing seasons, shallow or poorly developed soils, and stronger exposure may jointly restrict establishment and growth. This pattern is consistent with previous studies showing that the climatic responses and growth performance of *P. tabuliformis* vary along elevational gradients (Liu et al. 2016; Ning et al. 2023).

BIO18 reflects water supply during the warmest quarter and is an important moisture indicator affecting population maintenance and natural regeneration of *P. tabuliformis* . The consistent increase in suitability under low to moderate BIO18 values indicates that insufficient warm‐season precipitation forms an important moisture constraint. Although *P. tabuliformis* is generally considered drought tolerant, its growth and regeneration remain sensitive to water availability, particularly where warming increases atmospheric evaporative demand and soil‐water depletion (Chen et al. 2021; Zhao et al. 2021; Su et al. 2024). However, the divergence among models at moderate to high BIO18 values indicates greater uncertainty regarding the upper portion of the precipitation response. The RF and ensemble curves suggest diminishing benefits once a minimum moisture requirement has been met; the MaxEnt curve suggests a continued but gradually weakening positive response, and the GAM curve suggests a potential decline under relatively wet conditions. These differences may reflect algorithm‐specific smoothing, feature representation, or interactions with other environmental gradients.

Taken together, these findings also have implications for the climate‐change vulnerability of *P. tabuliformis* . The consistently high importance of BIO9, together with the contribution of BIO18, indicates that the species is sensitive to seasonal thermal and moisture conditions. Continued warming, increased evaporative demand, or reductions in warm‐season precipitation could therefore increase climatic stress in currently marginal habitats, particularly where water availability is already limited. However, because the present study evaluated habitat suitability only under current environmental conditions, these results should be interpreted as evidence of potential climatic sensitivity rather than as direct projections of future range change.

Within this current‐climate context, the identified response patterns and environmental constraints still provide useful guidance for niche management and habitat restoration of *P. tabuliformis* . However, the effects of these environmental factors may be more deeply associated with species functional traits (Peng, Ellison, and Davis 2025). Therefore, future studies could incorporate leaf, root, and physiological trait data to further reveal the adaptive mechanisms of *P. tabuliformis* under climate change.

### 4.3 Ecological and Management Implications

From an applied perspective, the spatial patterns and environmental constraints identified in this study have important implications for the conservation and management of *P. tabuliformis* . The ensemble model provided a synthesized spatial estimate derived from the three base models, with a total suitable‐habitat range that was more constrained than those predicted by RF and GAM. However, because its stability varied among evaluation metrics and its centroid uncertainty was intermediate, the ensemble should not be regarded as uniformly more stable than all individual models. Areas where mapped suitable habitats overlap across multiple model outputs may be treated as candidate areas for field verification. These recommendations should be viewed as a staged decision framework rather than as directly implementable prescriptions: candidate areas should first be evaluated for local site conditions, water availability, land use, and management feasibility, followed where appropriate by small‐scale pilot trials before broader implementation. In contrast, marginal areas or areas showing substantial disagreement among models should be prioritized for monitoring and field validation rather than large‐scale afforestation without sufficient field evidence (Ran et al. 2024).

The southern transitional distribution zone, particularly the Qinling Mountains, warrants particular attention in future management. Our spatial predictions identified this region as an important southern marginal zone of current potential suitability for *P. tabuliformis* . Previous tree‐ring studies in the Qinling–Funiu transition region have shown that the growth of *P. tabuliformis* is sensitive to seasonal temperature and water availability, with heat‐related water stress in May limiting radial growth at some sites (Chen et al. 2025; Peng, Peng, et al. 2025). Together, these findings support prioritizing the Qinling Mountains for long‐term monitoring of regeneration, radial growth, and stand condition. Therefore, management in the southern marginal zone should not simply focus on expanding afforestation area but should shift toward improving stand resilience. For example, water‐retention tending, appropriate density regulation, conversion to mixed conifer–broadleaf stands, and protection of natural regeneration can be adopted to reduce the risk of water deficit caused by increasing thermal stress (Marqués et al. 2022).

In contrast, in northern marginal and high‐elevation areas, expansion should not be based solely on the empirical perception that *P. tabuliformis* is tolerant to cold and drought. The results of this study showed that excessively low BIO9 values and excessively high DEM values both reduced habitat suitability, indicating that colder conditions or higher elevations do not necessarily correspond to more suitable habitats. In these areas, management practices should emphasize site matching, provenance selection, and small‐scale field trials rather than direct large‐scale expansion based only on model outputs. For afforestation and ecological restoration, model prediction maps should be understood as tools for risk identification rather than definitive planting boundaries. Core suitable areas can be prioritized for conservation and utilization, marginal areas require continuous monitoring, and uncertain areas should be evaluated through field validation and adaptive management. More broadly, climate‐adaptive management of *P. tabuliformis* should prioritize stand resilience over simple expansion of planting area, combine appropriate site and provenance matching with stand‐density and structural regulation, and adjust management interventions based on long‐term monitoring of regeneration, growth, and moisture stress.

### 4.4 Uncertainty in Model Predictions and Future Improvements

Although this study improved the rigor of model development and evaluation through variable screening, parameter optimization, and five‐fold spatial block cross‐validation, the uncertainties associated with the model predictions should still be interpreted with caution. First, although the occurrence records of *P. tabuliformis* were spatially thinned to reduce the effects of point clustering, records from public databases such as GBIF may still be affected by uneven survey effort, differences in accessibility, and historical sampling bias. In addition, no independent field‐survey records were available for external validation. Spatial thinning at a 1 km grid scale can reduce geographic duplication and sampling aggregation, but it cannot fully eliminate bias in geographic or environmental space, nor can it replace standardized field surveys (Bracho‐Estévanez et al. 2024; Ran et al. 2024). Moreover, retaining only one occurrence record within each 1 km × 1 km grid cell may have excluded some ecologically meaningful variation associated with fine‐scale habitat heterogeneity. However, because the environmental predictors were also standardized to a spatial resolution of 1 km, such within‐cell variation could not be explicitly represented in the current modeling framework. Future studies should assess the sensitivity of model predictions to alternative thinning distances and incorporate finer‐resolution environmental predictors and independent field observations.

Second, the construction of pseudo‐absence points may influence model predictive performance. Differences in sampling extent, number of pseudo‐absence points, and spatial constraints can alter model discrimination results. Future studies could compare multiple pseudo‐absence generation strategies and incorporate bias‐correction methods to further improve model reliability (Barbet‐Massin et al. 2012; Rausell‐Moreno et al. 2025).

Third, because the statistical comparisons and fold‐bootstrap analyzes were based on only five outer spatial folds, their statistical power and uncertainty estimates were limited. Nonsignificant pairwise tests should not be interpreted as evidence of model equivalence, and the reported confidence intervals reflect fold‐to‐fold variation under the selected spatial partitioning scheme rather than all sources of predictive uncertainty.

Fourth, although this study integrated multi‐source variables related to climate, topography, hydrology, and soil, some factors reflecting stand structure, biotic interactions, and human disturbance intensity were not included in the model, such as stand age structure, regeneration status, management practices, and pest or disease disturbances. Therefore, the current results mainly reveal broad‐scale associations between environmental gradients and the predicted current potential distribution of *P. tabuliformis* , while the representation of local‐scale ecological processes remains limited. Future studies integrating forest inventory data, remote‐sensing‐derived structural parameters, and long‐term monitoring records are expected to improve the ecological interpretability of the model (Peng, Ellison, and Davis 2025).

Fifth, the predicted suitable areas represent environmentally suitable potential habitat under the variables included in the model, rather than the actual occupied distribution of *P. tabuliformis* . High predicted suitability therefore does not necessarily indicate that the species currently occurs in a given location. Dispersal limitation, landscape fragmentation, land‐use conversion, barriers to establishment, local biotic interactions, and disturbance history may prevent the species from occupying some environmentally suitable areas. Accordingly, the predicted suitable‐habitat maps should be treated as tools for identifying candidate areas for field verification and management screening, rather than as confirmed occurrence maps or definitive afforestation boundaries (Sillero and Barbosa 2021). Future studies could integrate landscape connectivity, land‐use change, dispersal constraints, and independent forest‐inventory or field‐survey data to better distinguish potential habitat from the realized distribution.

Finally, this study did not project the future suitable habitat of *P. tabuliformis* under climate‐change scenarios. This decision was made because the present modeling framework incorporated not only bioclimatic variables but also hydrological, soil, land‐surface, and snow‐cover predictors. Although future projections could be conducted using only bioclimatic variables, doing so would require a predictor set different from that used in the current multi‐source model and would introduce additional uncertainty when comparing current and future suitability patterns. Therefore, the present study should be interpreted as an assessment of current potential habitat suitability. Future studies could develop a climate‐only or scenario‐compatible multi‐source framework to evaluate potential suitable‐habitat shifts under CMIP‐based climate scenarios.

Taken together, future work should focus on four priorities: independent field validation in regions of both model agreement and disagreement; sensitivity testing of spatial thinning and pseudo‐absence strategies under repeated spatial validation; integration of finer‐resolution forest‐structure, disturbance, connectivity, and land‐use data; and scenario‐compatible projections under CMIP‐based climate scenarios. These efforts would enable more rigorous evaluation of model transferability and provide a stronger basis for climate‐adaptive management of *P. tabuliformis*.

## 5 Conclusion

Based on distribution records of *Pinus tabuliformis* and multi‐source environmental variables, this study constructed MaxEnt, Random Forest (RF), Generalized Additive Model (GAM), and stacking ensemble models to predict the current potential suitable habitats of *P. tabuliformis* in China and identify the key environmental constraints. Three main findings emerged. First, seasonal hydrothermal conditions and topography were the major environmental constraints on habitat suitability. Mean temperature of the driest quarter (BIO9), elevation (DEM), and precipitation of the warmest quarter (BIO18) were consistently identified as the main predictors, with BIO9 ranking first across all four models. These findings support the first hypothesis that seasonal hydrothermal conditions and topography are major predictors of habitat suitability for *P. tabuliformis* .

Second, and importantly, the stacking ensemble did not outperform RF under five‐fold spatial block cross‐validation. RF achieved the highest mean AUC, TSS, and width‐normalized partial AUC, whereas the stacking ensemble produced AUC and TSS values close to those of RF. None of the pairwise differences between the ensemble and the individual models remained statistically significant after Holm correction. These results support the second hypothesis that stacking would not necessarily outperform individual models across all evaluation metrics under spatially independent validation. Therefore, the principal value of the stacking framework was not to uniformly improve predictive accuracy or stability, but to integrate probability predictions from structurally different algorithms, reduce reliance on any single model, and generate a synthesized spatial estimate.

Third, the predicted current potential suitable habitats were mainly distributed in the mountainous and hilly regions of North China, the Loess Plateau, the Qinling Mountains, and surrounding areas, supporting the third hypothesis. The ensemble predicted a total suitable area of 28.45 × 10^5^ km^2^, which was smaller than the estimates of RF and GAM but close to that of MaxEnt. It also retained a comparatively large highly suitable area. The ensemble should therefore be interpreted as providing a more spatially constrained estimate relative to RF and GAM, rather than as a uniformly conservative filter across all models and suitability classes.

Taken together, these results provide a spatially validated national‐scale assessment of the current potential distribution and major environmental constraints of *P. tabuliformis* , while also showing that structurally different models can yield similar discrimination performance but substantially different spatial predictions. This combination of environmental interpretation, spatial validation, and cross‐model comparison constitutes the main contribution of this study.

From an applied perspective, areas where suitable predictions overlap across multiple models should be prioritized for field verification. Only after local site conditions, land use, and management feasibility have been assessed should these areas be considered for seed‐source conservation, site‐specific restoration, or planting. In contrast, marginal areas or areas showing substantial disagreement among models should not be used for large‐scale afforestation without additional field evidence. Because our spatial predictions identified the Qinling Mountains as an important southern marginal transition zone, and previous tree‐ring studies have documented climate‐sensitive growth and heat‐related water stress in *P. tabuliformis* populations in this region (Chen et al. 2025; Peng, Peng, et al. 2025), we recommend prioritizing the Qinling Mountains for long‐term monitoring of regeneration, radial growth, and stand condition. As the models estimate potential environmental suitability rather than actual occupancy, the prediction maps should be used as screening and risk‐assessment tools rather than as definitive planting boundaries. Looking forward, future research should incorporate independent field validation, sensitivity analyzes of spatial thinning and pseudo‐absence strategies, finer‐resolution forest and landscape information, and scenario‐compatible projections under future climate scenarios. These efforts will help test the transferability of the current predictions, evaluate the potential climate‐change vulnerability of *P. tabuliformis* , and provide a stronger basis for climate‐adaptive management.

## Author Contributions

**Haodong Ding:** conceptualization (lead), data curation (lead), formal analysis (lead), methodology (lead), software (lead), validation (lead), visualization (lead), writing – original draft (lead), writing – review and editing (equal). **Jie Meng:** data curation (supporting), investigation (supporting), validation (supporting), writing – review and editing (equal). **Yaru Zhang:** data curation (supporting), formal analysis (supporting), visualization (supporting), writing – review and editing (equal). **Ying Yang:** data curation (supporting), investigation (supporting), validation (supporting), writing – review and editing (equal). **Wei Deng:** formal analysis (supporting), methodology (supporting), software (supporting), validation (supporting), writing – review and editing (equal). **Duanyang Xu:** conceptualization (supporting), funding acquisition (lead), project administration (lead), resources (lead), supervision (lead), writing – review and editing (equal).

## Funding

This work was supported by Development and Demonstration of an Integrated Ecosystem Restoration Effectiveness Assessment System Based on Multi‐Dimensional Vegetation Information Fusion (Grant 2024YFF1308105), Eco‐hydrology Evolution and Ecological Water Adaptation Pattern in the Aeolian Sand Zone of China (Grant 2024YFF1306301), and Young Top‐notch Talent of the Fifth Batch of Forestry and Grassland Science and Technology Innovation (Grant 2025132024).

## Conflicts of Interest

The authors declare no conflicts of interest.

## Supporting information


**Table S1:** Fold‐specific optimal hyperparameters for the base learners and stacking meta‐learner.
**Table S2:** Computational environment, hardware specifications, runtime, software, and package versions used in this study.
**Table S3:** Pairwise comparisons of model performance between the stacking ensemble and the individual models across five held‐out spatial folds.
**Table S4:** Pairwise and overall spatial overlap among habitat suitability predictions from RF, MaxEnt, GAM, and the stacking ensemble model.
**Table S5:** Sensitivity of predicted habitat areas to alternative suitability‐class thresholds for RF, MaxEnt, GAM, and the ensemble model.

## Data Availability

The species occurrence records of *Pinus tabuliformis* used in this study were obtained from the Global Biodiversity Information Facility (GBIF). Environmental variables were derived from publicly available datasets, including WorldClim, MERIT Hydro, Global Solar Atlas, EarthEnv, FAO soil data, and other open data sources listed in Table 1. The processed data and model outputs supporting the findings of this study are openly available in Zenodo at https://doi.org/10.5281/zenodo.19888282.

## References

[ece374290-bib-0001] Allouche, O. , A. Tsoar , and R. Kadmon . 2006. “Assessing the Accuracy of Species Distribution Models: Prevalence, Kappa and the True Skill Statistic (TSS).” Journal of Applied Ecology 43, no. 6: 1223–1232. 10.1111/j.1365-2664.2006.01214.x.

[ece374290-bib-0002] Araújo, M. B. , and M. New . 2007. “Ensemble Forecasting of Species Distributions.” Trends in Ecology & Evolution 22, no. 1: 42–47. 10.1016/j.tree.2006.09.010. 17011070

[ece374290-bib-0003] Barbet‐Massin, M. , F. Jiguet , C. H. Albert , and W. Thuiller . 2012. “Selecting Pseudo‐Absences for Species Distribution Models: How, Where and How Many?” Methods in Ecology and Evolution 3, no. 2: 327–338. 10.1111/j.2041-210X.2011.00172.x.

[ece374290-bib-0004] Bi, Y. F. , J. C. Xu , Q. H. Li , et al. 2013. “Applying BioMod for Model‐Ensemble in Species Distributions: A Case Study for Tsuga Chinensis in China.” Plant Diversity and Resources 35, no. 5: 647–655. 10.7677/ynzwyj201312127.

[ece374290-bib-0005] Bracho‐Estévanez, C. A. , S. Arenas‐Castro , J. P. González‐Varo , and P. González‐Moreno . 2024. “Spatially Explicit Metrics Improve the Evaluation of Species Distribution Models Facing Sampling Biases.” Ecological Informatics 84: 102916. 10.1016/j.ecoinf.2024.102916.

[ece374290-bib-0006] Breiman, L. 1996. “Stacked Regressions.” Machine Learning 24, no. 1: 49–64. 10.1007/BF00117832.

[ece374290-bib-0007] Breiman, L. 2001. “Random Forests.” Machine Learning 45, no. 1: 5–32. 10.1023/A:1010933404324.

[ece374290-bib-0008] Chen, M. , X. Zhang , M. Li , J. Zhang , and Y. Cao . 2021. “Climate‐Growth Pattern of *Pinus tabulaeformis* Plantations and Their Resilience to Drought Events in the Loess Plateau.” Forest Ecology and Management 499: 119642. 10.1016/j.foreco.2021.119642.

[ece374290-bib-0009] Chen, Q. , N. Liu , G. Bao , et al. 2025. “Growth Response of *Pinus tabuliformis* and *Abies fargesii* to Climate Factors in Southern Slope of Central Qinling Mountains of China.” Forests 16, no. 2: 232. 10.3390/f16020232.

[ece374290-bib-0010] Cobos, M. E. , A. T. Peterson , N. Barve , and L. Osorio‐Olvera . 2019. “Kuenm: An R Package for Detailed Development of Ecological Niche Models Using Maxent.” PeerJ 7: e6281. 10.7717/peerj.6281. 30755826 PMC6368831

[ece374290-bib-0011] Cutler, D. R. , T. C. Edwards Jr. , K. H. Beard , et al. 2007. “Random Forests for Classification in Ecology.” Ecology 88, no. 11: 2783–2792. 10.1890/07-0539.1. 18051647

[ece374290-bib-0012] Dormann, C. F. , J. Elith , S. Bacher , et al. 2013. “Collinearity: A Review of Methods to Deal With It and a Simulation Study Evaluating Their Performance.” Ecography 36: 27–46. 10.1111/j.1600-0587.2012.07348.x.

[ece374290-bib-0013] Elith, J. , S. J. Phillips , T. Hastie , M. Dudík , Y. E. Chee , and C. J. Yates . 2011. “A Statistical Explanation of MaxEnt for Ecologists.” Diversity and Distributions 17, no. 1: 43–57. 10.1111/j.1472-4642.2010.00725.x.

[ece374290-bib-0014] Hao, T. , J. Elith , G. Guillera‐Arroita , and J. J. Lahoz‐Monfort . 2019. “A Review of Evidence About Use and Performance of Species Distribution Modelling Ensembles Like BIOMOD.” Diversity and Distributions 25, no. 5: 839–852. 10.1111/ddi.12892.

[ece374290-bib-0015] Hao, T. , J. Elith , J. J. Lahoz‐Monfort , and G. Guillera‐Arroita . 2020. “Testing Whether Ensemble Modelling Is Advantageous for Maximising Predictive Performance of Species Distribution Models.” Ecography 43, no. 4: 549–558. 10.1111/ecog.04890.

[ece374290-bib-0016] Hastie, T. J. , and R. J. Tibshirani . 2017. Generalized Additive Models. 1st ed. Routledge. 10.1201/9780203753781. 8548102

[ece374290-bib-0017] Koldasbayeva, D. , and A. Zaytsev . 2025. “Foundation for Unbiased Cross‐Validation of Spatio‐Temporal Models for Species Distribution Modeling.” Ecological Informatics 92: 103521. 10.1016/j.ecoinf.2025.103521.

[ece374290-bib-0018] Li, H. , Z. H. Zhang , and S. Q. Jian . 2023. “Simulation Analysis of Potential Suitable Distribution Area of *Pinus tabuliformis* in Yiluo River Basin Based on MaxEnt Model.” Journal of Zhejiang Forestry Science and Technology 43, no. 1: 1–8. 10.3969/j.issn.1001-3776.2023.01.001.

[ece374290-bib-0019] Li, Z. , C. Wang , G. Gao , et al. 2026. “Climate Warming Induced Pervasive Growth Decline in Chinese Pine Populations of the Loess Plateau, China.” Frontiers in Plant Science 17: 1749887. 10.3389/fpls.2026.1749887. 41815426 PMC12971888

[ece374290-bib-0020] Liu, X. , C. Gao , Q. Su , Y. Zhang , and Y. Song . 2016. “Altitudinal Trends in δ13C Value, Stomatal Density and Nitrogen Content of *Pinus tabuliformis* Needles on the Southern Slope of the Middle Qinling Mountains, China.” Journal of Mountain Science 13, no. 6: 1066–1077. 10.1007/s11629-015-3532-8.

[ece374290-bib-0021] Liu, Y. , and H. S. Wang . 2024. “Effects of Climate Change on Distribution of Suitable Area of *Pinus tabuliformis* Plantation in China.” Journal of Beijing Forestry University 46, no. 6: 82–92. 10.12171/j.1000-1522.20230072.

[ece374290-bib-0022] Ma, D. , H. Deng , Y. Yin , S. Wu , and D. Zheng . 2019. “Sensitivity of Arid/Humid Patterns in China to Future Climate Change Under a High‐Emissions Scenario.” Journal of Geographical Sciences 29: 29–48. 10.1007/s11442-019-1582-5.

[ece374290-bib-0023] Marmion, M. , M. Parviainen , M. Luoto , R. K. Heikkinen , and W. Thuiller . 2009. “Evaluation of Consensus Methods in Predictive Species Distribution Modelling.” Diversity and Distributions 15, no. 1: 59–69. 10.1111/j.1472-4642.2008.00491.x.

[ece374290-bib-0024] Marqués, L. , D. M. P. Peltier , J. J. Camarero , et al. 2022. “Disentangling the Legacies of Climate and Management on Tree Growth.” Ecosystems 25, no. 1: 215–235. 10.1007/s10021-021-00650-8. 35210936 PMC8827397

[ece374290-bib-0025] Martínez‐Minaya, J. , M. Cameletti , D. Conesa , and M. G. Pennino . 2018. “Species Distribution Modeling: A Statistical Review With Focus in Spatio‐Temporal Issues.” Stochastic Environmental Research and Risk Assessment 32, no. 11: 3227–3244. 10.1007/s00477-018-1548-7.

[ece374290-bib-0026] Melo, F. 2013. “Receiver Operating Characteristic (ROC) Curve.” In Encyclopedia of Systems Biology, edited by W. Dubitzky , O. Wolkenhauer , K.‐H. Cho , and H. Yokota , 1818–1823. Springer. 10.1007/978-1-4419-9863-7_242.

[ece374290-bib-0027] Merow, C. , M. J. Smith , and J. A. Silander Jr. 2013. “A Practical Guide to MaxEnt for Modeling Species' Distributions: What It Does, and Why Inputs and Settings Matter.” Ecography 36, no. 10: 1058–1069. 10.1111/j.1600-0587.2013.07872.x.

[ece374290-bib-0028] Montoya‐Jiménez, J. C. , J. R. Valdez‐Lazalde , G. Ángeles‐Pérez , H. M. De los Santos‐Posadas , and G. Cruz‐Cárdenas . 2025. “Evaluation of the Configuration of Three Algorithms to Model the Potential Distribution of Forest Species.” Ecosistemas y Recursos Agropecuarios 12, no. 2: e4127. 10.19136/era.a12n2.4127.

[ece374290-bib-0029] Ning, P. , M. Zhang , T. Bai , et al. 2023. “Dendroclimatic Response of *Pinus tabuliformis* Carr. Along an Altitudinal Gradient in the Warm Temperate Region of China.” Frontiers in Plant Science 14: 1147229. 10.3389/fpls.2023.1147229. 37063178 PMC10097970

[ece374290-bib-0030] Norberg, A. , N. Abrego , F. G. Blanchet , et al. 2019. “A Comprehensive Evaluation of Predictive Performance of 33 Species Distribution Models at Species and Community Levels.” Ecological Monographs 89, no. 3: e01370. 10.1002/ecm.1370.

[ece374290-bib-0031] Oeser, J. , D. Zurell , F. Mayer , et al. 2024. “The Best of Two Worlds: Using Stacked Generalisation for Integrating Expert Range Maps in Species Distribution Models.” Global Ecology and Biogeography 33, no. 12: e13911. 10.1111/geb.13911.

[ece374290-bib-0032] Peng, K. , J. Peng , J. Li , et al. 2025. “Regional Growth Response and Resilience of *Pinus tabuliformis* to Climate Change in the North–South Transition Zone, Central China.” Journal of Plant Ecology 18, no. 3: rtaf042. 10.1093/jpe/rtaf042.

[ece374290-bib-0033] Peng, S. , A. M. Ellison , and C. C. Davis . 2025. “Incorporating Responses of Traits to Changing Climates Into Species Distribution Models: A Path Forward.” New Phytologist 248: 38–49. 10.1111/nph.70402. 40716033

[ece374290-bib-0034] Peterson, A. T. , M. Papeş , and J. Soberón . 2008. “Rethinking Receiver Operating Characteristic Analysis Applications in Ecological Niche Modeling.” Ecological Modelling 213, no. 1: 63–72. 10.1016/j.ecolmodel.2007.11.008.

[ece374290-bib-0035] Peterson, A. T. , Y. Yao , M. E. Cobos , and X. Xiao . 2024. “Correlative Ecological Niche Model Applications to Predicting Landscape‐Scale Woody Plant Encroachment in Kansas Tallgrass Prairie Systems.” PLoS One 19, no. 6: e0305168. 10.1371/journal.pone.0305168. 38870187 PMC11175484

[ece374290-bib-0036] Phillips, S. J. , R. P. Anderson , and R. E. Schapire . 2006. “Maximum Entropy Modeling of Species Geographic Distributions.” Ecological Modelling 190, no. 3: 231–259. 10.1016/j.ecolmodel.2005.03.026.

[ece374290-bib-0037] Phillips, S. J. , and M. Dudík . 2008. “Modeling of Species Distributions With Maxent: New Extensions and a Comprehensive Evaluation.” Ecography 31, no. 2: 161–175. 10.1111/j.0906-7590.2008.5203.x.

[ece374290-bib-0038] Phillips, S. J. , M. Dudík , J. Elith , et al. 2009. “Sample Selection Bias and Presence‐Only Distribution Models: Implications for Background and Pseudo‐Absence Data.” Ecological Applications 19, no. 1: 181–197. 10.1890/07-2153.1. 19323182

[ece374290-bib-0039] Qin, A. , B. Liu , Q. Guo , et al. 2017. “MaxEnt Modeling for Predicting Impacts of Climate Change on the Potential Distribution of *Thuja sutchuenensis* Franch, an Extremely Endangered Conifer From Southwestern China.” Global Ecology and Conservation 10: 139–146. 10.1016/j.gecco.2017.02.004.

[ece374290-bib-0040] Qu, K. , C. Zhou , D. Liu , et al. 2025. “Constans‐Like and Short Vegetative Phase‐Like Genes Coordinately Modulate Terminal Flower 2 to Control Dormancy Transitions in *Pinus tabuliformis* .” Plant, Cell & Environment 48: 3066–3084. 10.1111/pce.15313. 39676713

[ece374290-bib-0041] Ran, W. , J. Chen , Y. Zhao , et al. 2024. “Global Climate Change‐Driven Impacts on the Asian Distribution of Limassolla Leafhoppers, With Implications for Biological and Environmental Conservation.” Ecology and Evolution 14, no. 7: e70003. 10.1002/ece3.70003. 39026963 PMC11257772

[ece374290-bib-0042] Rausell‐Moreno, A. , N. Galiana , B. Naimi , and M. B. Araújo . 2025. “Improving Species Distribution Models by Optimising Background Points: Impacts on Current and Future Climate Projections.” Ecological Modelling 507: 111177. 10.1016/j.ecolmodel.2025.111177.

[ece374290-bib-0043] Richardson, E. , R. Trevizani , J. A. Greenbaum , H. Carter , M. Nielsen , and B. Peters . 2024. “The Receiver Operating Characteristic Curve Accurately Assesses Imbalanced Datasets.” Patterns 5: 100994. 10.1016/j.patter.2024.100994. 39005487 PMC11240176

[ece374290-bib-0044] Sillero, N. , and A. M. Barbosa . 2021. “Common Mistakes in Ecological Niche Models.” International Journal of Geographical Information Science 35, no. 2: 213–226. 10.1080/13658816.2020.1798968.

[ece374290-bib-0045] Smith, A. B. , and M. J. Santos . 2020. “Testing the Ability of Species Distribution Models to Infer Variable Importance.” Ecography 43, no. 12: 1801–1813. 10.1111/ecog.05317.

[ece374290-bib-0046] Su, J. R. , S. C. Xiao , X. M. Peng , C. W. Che , and P. Zhao . 2024. “Regional Differentiation of Radial Growth to Climate Response of Chinese Pine (*Pinus tabulaeformis*).” Journal of Desert Research 44, no. 5: 60–72. 10.7522/j.issn.1000-694X.2024.00034.

[ece374290-bib-0047] Syphard, A. D. , and J. Franklin . 2009. “Differences in Spatial Predictions Among Species Distribution Modeling Methods Vary With Species Traits and Environmental Predictors.” Ecography 32, no. 6: 907–918. 10.1111/j.1600-0587.2009.05883.x.

[ece374290-bib-0048] Wang, A. J. , D. Y. Lu , G. S. Zhang , et al. 2021. “Potential Distribution of *Juniperus sabina* Under Climate Change in Eurasia Continent Based on MaxEnt Model.” Scientia Silvae Sinica 57, no. 8: 43–55. 10.11707/j.1001-7488.20210805.

[ece374290-bib-0049] Wolpert, D. H. 1992. “Stacked Generalization.” Neural Networks 5, no. 2: 241–259. 10.1016/S0893-6080(05)80023-1.

[ece374290-bib-0050] Wood, S. N. 2017. Generalized Additive Models: An Introduction With R. 2nd ed. Chapman and Hall/CRC. 10.1201/9781315370279.

[ece374290-bib-0051] Xie, S. , G. Yin , W. Wei , Q. Sun , and Z. Zhang . 2022. “Spatial–Temporal Change in Paddy Field and Dryland in Different Topographic Gradients: A Case Study of China During 1990–2020.” Land 11: 1851. 10.3390/land11101851.

[ece374290-bib-0052] Yang, H. , Y. Song , Y. Pang , H. Kang , Y. Xue , and D. Wang . 2023. “Driving Factors of Chinese Pine Population Distribution in the Ridge Habitats of the Southern Slope of the Mid‐Qinling Mountains, China.” Forests 14: 2252. 10.3390/f14112252.

[ece374290-bib-0053] Yee, T. W. , and N. D. Mitchell . 1991. “Generalized Additive Models in Plant Ecology.” Journal of Vegetation Science 2, no. 5: 587–602. 10.2307/3236170.

[ece374290-bib-0054] Zhang, R. , F. Wang , J. Zheng , et al. 2026. “Effective Chilling Temperatures for Dormancy Release in Extratropical Forest Trees Increase From Cold to Warm Regions.” Proceedings of the National Academy of Sciences of the United States of America 123, no. 2: e2531077123. 10.1073/pnas.2531077123. 41499405 PMC12798943

[ece374290-bib-0055] Zhang, X. , S. Zheng , and Z. Shangguan . 2006. “Nutrient Distributions and Bio‐Cycle Characteristics in Both Natural and Artificial *Pinus tabulaeformis* Carr. Forests in Hilly Loess Regions.” Acta Ecologica Sinica 26, no. 2: 373–382. 10.1016/S1872-2032(06)60008-5.

[ece374290-bib-0056] Zhao, Y. , L. X. Cai , Y. T. Jin , J. X. Li , D. Cui , and Z. J. Chen . 2021. “Warming‐Drying Climate Intensifies the Restriction of Moisture on Radial Growth of *Pinus tabuliformis* Plantation in Semi‐Arid Area of Northeast China.” Chinese Journal of Applied Ecology 32, no. 10: 3459–3467. 10.13287/j.1001-9332.202110.034. 34676706

[ece374290-bib-0057] Zhao, Z. , N. Xiao , M. Shen , and J. Li . 2022. “Comparison Between Optimized MaxEnt and Random Forest Modeling in Predicting Potential Distribution: A Case Study With *Quasipaa boulengeri* in China.” Science of the Total Environment 842: 156867. 10.1016/j.scitotenv.2022.156867. 35752245

[ece374290-bib-0058] Zhu, G. P. , J. Y. Fan , M. L. Wang , M. Chen , and H. J. Qiao . 2017. “The Importance of the Shape of Receiver Operating Characteristic (ROC) Curve in Ecological Niche Model Evaluation: Case Study of *Hyphantria cunea* .” Journal of Biosafety 26, no. 3: 184–190. 10.3969/j.issn.2095-1787.2017.03.002.

