# Supplementary material for: Predicting the Current Potential Suitable Habitat of Pinus tabuliformis in China Using Multi‐Source Environmental Data and Ensemble Learning Models
Source: Ecol Evol. 2026 Sep 10;16(9):e74290. doi: 10.1002/ece3.74290 (PMC13561856; doi:10.1002/ece3.74290)
Supplement: Supplementary file 1 [file ECE3-16-e74290-s001.docx]

Supplementary Table S1. Fold-specific optimal hyperparameters for the base learners and stacking meta-learner.

| **Outer spatial fold** | **RF**  **max_depth** | **RF**  **min_samples_leaf** | **GAM**  **n_splines** | **GAM**  **λ** | **MaxEnt**  **FC** | **MaxEnt**  **RM** | **Ensemble C** |
| --- | --- | --- | --- | --- | --- | --- | --- |
| Fold 1 | Unrestricted | 1 | 6 | 14.657 | LQHP | 1.5 | 3.162 |
| Fold 2 | Unrestricted | 1 | 6 | 1.153 | LQHP | 1 | 3.162 |
| Fold 3 | 10 | 5 | 8 | 4.928 | LQHP | 3.5 | 10 |
| Fold 4 | 10 | 5 | 9 | 3.511 | LQHP | 2 | 1 |
| Fold 5 | 5 | 3 | 7 | 1.173 | LQHP | 1 | 31.623 |

Note: For RF, the number of trees and max_features were fixed at 500 and 4, respectively. For GAM, an intercept and quadratic penalized B-spline basis functions were used in all folds. LQHP denotes linear, quadratic, hinge, and product feature classes, and RM denotes the MaxEnt regularization multiplier. For the stacking ensemble, C denotes the inverse regularization-strength parameter of the logistic-regression meta-learner; larger C values correspond to weaker regularization. The value of C was selected from 13 logarithmically spaced candidate values ranging from 10^-3^ to 10^3^ using internal cross-validated AUC. All hyperparameters were optimized independently within each outer spatial training dataset.

Supplementary Table S2. Computational environment, hardware specifications, runtime, software, and package versions used in this study.

| **Component** | **Item** | **Version/specification** | **Purpose in this study** |
| --- | --- | --- | --- |
| Operating system | Windows | Windows10, build 19045 | Computational environment |
| Processor | Intel Core i7-14700 | 20 physical cores, 28 logical threads | Model training, hyperparameter optimization, and spatial prediction |
| Memory | RAM | 32 GB | Model fitting and raster processing |
| Runtime | Full five-fold modeling workflow | Approximately 36 h | Hyperparameter tuning, model fitting, held-out evaluation, and full-extent raster prediction |
| Programming language | Python | 3.12.6 | Main workflow implementation |
| Programming language | R | 4.5.1 | MaxEnt implementation, pseudo-absence generation, and spatial block construction |
| Python package | numpy | 1.26.4 | Numerical computation |
| Python package | pandas | 2.2.3 | Tabular data processing |
| Python package | geopandas | 1.0.1 | Spatial point data processing |
| Python package | rasterio | 1.3.10 | Raster reading, writing, and spatial prediction |
| Python package | rasterstats | 0.19.0 | Extraction of raster values at occurrence and pseudo-absence points |
| Python package | shapely | 2.0.4 | Geometric object construction and spatial processing |
| Python package | scikit-learn | 1.5.2 | RF modeling, stacking ensemble construction, logistic regression, internal cross-validation, hyperparameter optimization, and model evaluation |
| Python package | pyGAM | 0.9.1 | Logistic GAM modeling |
| Python package | statsmodels | 0.14.3 | Variance inflation factor calculation |
| Python package | scipy | 1.13.1 | Partial AUC calculation and statistical computation |
| Python package | matplotlib | 3.8.4 | Plotting ROC curves, variable importance, and response curves |
| Python package | rpy2 | 3.5.11 | Python–R interface for calling R packages |
| Python package | pyproj | 3.6.1 | Geodesic raster-cell area calculation for spatial-overlap analysis |
| R package | dismo | 1.3-16 | Interface for fitting the Java implementation of MaxEnt |
| R package | raster | 3.6-32 | Raster data handling in R |
| R package | rJava | 1.0-11 | Java interface required for MaxEnt |
| R package | sp | 2.2-0 | Spatial object processing in R |
| R package | biomod2 | 4.2.6.2 | Pseudo-absence generation |
| R package | blockCV | 3.2.0 | Spatial autocorrelation range estimation and five-fold spatial block cross-validation |
| GIS software | ArcGIS Pro | 3.3.2 | Habitat suitability reclassification, area statistics, centroid analysis |
| Modeling software | MaxEnt | 3.4.3 | Maximum entropy species distribution modeling |

Supplementary Table S3. Pairwise comparisons of model performance between the stacking ensemble and the individual models across five held-out spatial folds.

| **Metric** | **Comparison** | **Paired t-test**  **P** | **Holm-adjusted**  **P** | **Wilcoxon signed-rank**  **P** | **Holm-adjusted**  **P** |
| --- | --- | --- | --- | --- | --- |
| AUC | Ensemble vs RF | 0.784642588 | 1 | 1 | 1 |
| AUC | Ensemble vs MaxEnt | 0.093767359 | 0.750138871 | 0.0625 | 0.5625 |
| AUC | Ensemble vs GAM | 0.153826033 | 1 | 0.0625 | 0.5625 |
| TSS | Ensemble vs RF | 0.755372657 | 1 | 1 | 1 |
| TSS | Ensemble vs MaxEnt | 0.187676897 | 1 | 0.125 | 0.625 |
| TSS | Ensemble vs GAM | 0.31281889 | 1 | 0.375 | 1 |
| Partial AUC (FPR 0–0.1) | Ensemble vs RF | 0.485671418 | 1 | 0.625 | 1 |
| Partial AUC (FPR 0–0.1) | Ensemble vs MaxEnt | 0.029503303 | 0.265529726 | 0.0625 | 0.5625 |
| Partial AUC (FPR 0–0.1) | Ensemble vs GAM | 0.160429743 | 1 | 0.0625 | 0.5625 |

Note: Paired-sample t-tests were used as the primary comparisons, with Wilcoxon signed-rank tests as a nonparametric robustness check; P values were adjusted using the Holm method across the nine planned comparisons (three metrics × three base models), and no comparison remained statistically significant after adjustment (adjusted P > 0.05).

Supplementary Table S4. Pairwise and overall spatial overlap among habitat suitability predictions from RF, MaxEnt, GAM, and the stacking ensemble model.

| **Model comparison** | **Total suitable intersection (×10⁵ km²)** | **Jaccard index (≥0.2)** | **Highly suitable intersection (×10⁵ km²)** | **Jaccard index (≥0.6)** |
| --- | --- | --- | --- | --- |
| RF vs. MaxEnt | 24.74 | 0.651 | 6.17 | 0.450 |
| RF vs. GAM | 28.37 | 0.725 | 12.56 | 0.621 |
| RF vs. Ensemble | 27.45 | 0.774 | 13.20 | 0.700 |
| MaxEnt vs. GAM | 25.38 | 0.704 | 6.24 | 0.321 |
| MaxEnt vs. Ensemble | 25.00 | 0.786 | 6.40 | 0.344 |
| GAM vs. Ensemble | 27.14 | 0.789 | 16.64 | 0.783 |
| Four-model | 23.08 | 0.567 | 6.05 | 0.283 |

Note: Total suitable habitat was defined as suitability ≥ 0.2 and highly suitable habitat as suitability ≥ 0.6. For pairwise model comparisons, the Jaccard index was calculated as the intersection area divided by the union area. For the four-model comparison, the reported value represents the area jointly classified as suitable by all four models divided by the union area classified as suitable by at least one model. Values closer to 1 indicate greater spatial overlap.

Supplementary Table S5. Sensitivity of predicted habitat areas to alternative suitability-class thresholds for RF, MaxEnt, GAM, and the ensemble model.

| **Threshold scheme** | **Thresholds** | **Model** | **Low** | **Moderate** | **High** | **Total suitable** | **Δ Total (%)** | **Δ High (%)** |
| --- | --- | --- | --- | --- | --- | --- | --- | --- |
| Lower | 0.15/0.35/0.55 | RF | 13.77 | 9.25 | 15.96 | 38.98 | 13.17 | 18.42 |
|  |  | MaxEnt | 11.71 | 11.24 | 8.82 | 31.77 | 12.09 | 37.73 |
|  |  | GAM | 10.17 | 5.68 | 20.62 | 36.47 | 10.29 | 6.84 |
|  |  | Ensemble | 7.55 | 3.89 | 19.52 | 30.95 | 8.81 | 5.01 |
| Baseline | 0.20/0.40/0.60 | RF | 11.35 | 9.62 | 13.48 | 34.44 | — | — |
|  |  | MaxEnt | 11.08 | 10.85 | 6.4 | 28.34 | — | — |
|  |  | GAM | 8.35 | 5.42 | 19.3 | 33.07 | — | — |
|  |  | Ensemble | 6.12 | 3.74 | 18.59 | 28.45 | — | — |
| Higher | 0.25/0.45/0.65 | RF | 9.61 | 9.8 | 11.12 | 30.53 | −11.36 | −17.47 |
|  |  | MaxEnt | 11.34 | 10.12 | 4.02 | 25.48 | −10.07 | −37.22 |
|  |  | GAM | 7.02 | 5.36 | 17.91 | 30.29 | −8.40 | −7.21 |
|  |  | Ensemble | 4.93 | 3.74 | 17.61 | 26.28 | −7.61 | −5.25 |

Note: Habitat suitability was classified using three threshold schemes: lower (0.15, 0.35, and 0.55), baseline (0.20, 0.40, and 0.60), and higher (0.25, 0.45, and 0.65). Low, moderate, and high suitability correspond to the three intervals defined by each threshold scheme, and total suitable area is the sum of these three classes. Δ Total and Δ High represent percentage changes in total suitable area and highly suitable area, respectively, relative to the baseline scheme. Area values are expressed as ×10⁵ km².
